# Validation of a microRNA target site polymorphism in *H3F3B* that is potentially associated with a broad schizophrenia phenotype

**DOI:** 10.1371/journal.pone.0194233

**Published:** 2018-03-12

**Authors:** William Manley, Michael P. Moreau, Marco Azaro, Stephen K. Siecinski, Gillian Davis, Steven Buyske, Veronica Vieland, Anne S. Bassett, Linda Brzustowicz

**Affiliations:** 1 Department of Genetics, Rutgers University, Piscataway, NJ, United States of America; 2 Department of Statistics & Biostatistics, Rutgers University, Piscataway, NJ, United States of America; 3 Battelle Center for Mathematical Medicine, The Research Institute at Nationwide Children’s Hospital, Columbus, Ohio, United States of America; 4 Clinical Genetics Research Program, Centre for Addiction and Mental Health, Toronto, ON, Canada; Universitat des Saarlandes, GERMANY

## Abstract

Despite much progress, few genetic findings for schizophrenia have been assessed by functional validation experiments at the molecular level. We previously reported evidence for genetic linkage of broadly defined schizophrenia to chromosome 17q25 in a sample of 24 multiplex families. 2,002 SNPs under this linkage peak were analyzed for evidence of linkage disequilibrium using the posterior probability of linkage (PPL) framework. SNP rs1060120 produced the strongest evidence for association, with a PPLD|L score of 0.21. This SNP is located within the 3'UTR of the histone gene *H3F3B* and colocalizes with potential gene target miR-616. A custom miRNA target prediction program predicted that the binding of miR-616 to *H3F3B* transcripts would be altered by the allelic variants of rs1060120. We used dual luciferase assays to experimentally validate this interaction. The rs1060120 A allele significantly reduced luciferase expression, indicating a stronger interaction with miR-616 than the G allele (p = 0.000412). These results provide functional validation that this SNP could alter schizophrenia epigenetic mechanisms thereby contributing to schizophrenia-related disease risk.

## Introduction

Schizophrenia is a complex disorder that is believed to be caused by the interaction of multiple genetic risk factors, within and between individuals. Environmental factors may also play a role. Considerable advances have been made in schizophrenia genetics. This includes recent work by the Psychiatric Genomics Consortium (PGC) that has reported 108 independent associations with common genetic variants (SNPs), 83 of which were newly implicated in schizophrenia, from studies involving over 35,000 cases [1]. This work has also indicated a high genetic correlation between schizophrenia and bipolar disorder and a moderate genetic correlation between schizophrenia and major depressive disorder[2]. In other studies, both common risk alleles of low effect size and rare mutations of high effect size have been shown to contribute to schizophrenia and to other psychiatric disorders [2–4]. These risk variants appear to cluster in functional gene sets involving genes that cause alterations in the regulation of gene expression in the brain[5–6], genes important for calcium channels and glutamatergic synapses [7–9], and genes that alter the expression profiles of miRNAs[10–17].

miRNAs are small endogenous RNA molecules that are able to bind to specific regions of the 3’ UTR of coding mRNAs thereby decreasing expression of their protein product [18]. This is relevant since miRNAs are also known to have important roles in brain development and the development of schizophrenia and other psychiatric disorders [10,12,13,18,19]. Postmortem studies have shown that miRNAs are differentially expressed in individuals with schizophrenia [11, 14] and genome-wide association studies GWAS have shown that miRNAs contribute to the variants in gene expression found in individuals with schizophrenia [20]. These differentially expressed miRNAs have been shown to have altered targeting ability for specific alleles associated with increased risk of illness [16,21]. Here we present evidence that the rs1060120 allele is associated with a broad schizophrenia spectrum phenotype and alters the function of a miRNA target site within the 3’ UTR of *H3F3B*.

In 2000 we published a traditional parametric linkage analysis using genome-wide microsatellite markers to search for linkage to schizophrenia and related phenotypes within a sample of 22 Canadian extended families recruited for study because multiple relatives were clinically diagnosed with schizophrenia or schizoaffective disorder [22]. We subsequently re-analyzed the genome scan data using a Bayesian linkage analysis technique, the posterior probability of linkage, which has the advantage of retaining the power of parametric linkage analysis yet being model-free, and with notable enhanced signal-to-noise ratio when compared to traditional parametric analysis [23]. Both analyses produced strong evidence of linkage to chromosome 1q23 under a narrow definition of affection (schizophrenia or chronic schizoaffective disorder), which we have extensively investigated [22,24–27]. The PPL analysis also produced clear evidence for a linkage peak on 17q25, but only under a much broader definition of affection that included a number of schizophrenia-spectrum diagnoses.

A meta-analysis of schizophrenia linkage studies identified a suggestive level peak on 17q, within 4 Mb of the linkage peak in our sample [28]. While this meta-analysis primarily considered studies focusing on a narrow definition of schizophrenia (schizophrenia and schizoaffective disorder), three of the 20 studies analyzed used a broader phenotypic definition of affection. Interestingly, the ranking of this 17q region decreased in a subsequent larger meta-analysis conducted by the same investigators, although in the expanded analysis only two of the 32 studies analyzed used a broader affection phenotype [29]. This same region of 17q was subsequently implicated in a sibpair linkage analysis of 175 families of Mexican and Central American ancestry; strong linkage signals were observed under both narrow (schizophrenia and schizoaffective disorder) and broad (narrow plus other psychotic disorders) phenotypes [30].

Given the evidence in our family sample for a locus related to a broad schizophrenia phenotype on chromosome 17, we conducted additional studies to identify and validate a functional variant under the linkage peak that exhibited strong linkage disequilibrium with our broad phenotype. Because of the importance of miRNAs in schizophrenia, we elected to interrogate the region under our linkage peak with SNPs predicted to alter miRNA targets in addition to the stock SNP content pulled from a dense GWAS mapping array. Here we present evidence that a SNP in the gene *H3F3B* is associated with a broad schizophrenia spectrum phenotype in our family sample and also alters the function of a miRNA target site within the 3’ UTR of the gene.

## Materials and methods

### Genetic association subjects

The subjects analyzed for this study are the same that were previously used in our association studies of *NOS1AP* [25]. The sample consisted of 24 Canadian families of Celtic (n = 23) or German (n = 1) descent, recruited for study because schizophrenic illness appeared to be segregating in a unilineal (one side of the family) autosomal dominant-like manner. After complete description of the study to the subjects, written informed consent was obtained. Protocols were approved by the Institutional Review Boards of Rutgers University, University of Toronto, and Centre for Addiction and Mental Health. The primary phenotype for this study was a broad schizophrenia spectrum which coded as affected 85 individuals with a diagnosis of schizophrenia or chronic schizoaffective disorder as well as 40 individuals with the spectrum diagnoses of non-affective psychotic disorder, schizotypal personality disorder, or paranoid personality disorder. One hundred and ninety two individuals were coded as unaffected. For a secondary analysis using a narrow phenotype, only individuals with a diagnosis of schizophrenia or chronic schizoaffective disorder were coded as affected, with other assessed family members coded unaffected. DNA samples were available on 332 subjects. Subjects with available DNA but no assessment data were coded phenotype unknown. The ascertainment and assessment procedures and composition of the sample have been previously described in greater detail [22,25,31,32].

### SNP selection and genotyping

The linkage peak previously identified on chromosome 17q25 [23] was bounded by D17S1301 and 17qter, corresponding to position 74,684,647 to 83,257,441 on GRCh38. We identified 2,002 SNPs from the Affymetrix 6.0 Array as residing under this linkage peak. We also searched for SNPs under the linkage peak that were predicted to alter miRNA binding sites (MirSNPs). To do this, a custom miRNA target prediction program was developed that followed the procedure embodied by rna22 [33] with some modifications. rna22 was considered an appealing exemplar since it does not rely on cross-species conservation to prioritize candidate targets. Instead it extracts conserved patterns from a set of mature miRNAs and scans 3'UTR transcripts for regions that have a significant enrichment of these derived patterns. Whereas rna22 uses Release 3.0 of Mirbase [34] as a pan-species source of mature miRNAs (comprising 6 species), our implementation utilizes human-specific miRNAs obtained from Release 12.0 (Sep 2009). In addition, our 3'UTR substrate sequences were obtained from AceView [35] (Human Apr07 release). Not only is this a well-curated and comprehensive source of transcript sequences but the authors note that the intron boundaries defined by the AceView assembly are considered to be very reliable and delineate the 3'UTR regions with high confidence. Furthermore, these 3'UTR sequences were substituted with SNPs obtained from build 129 of dbSNP [36]. In particular, SNPs that fell within the bounds of a given 3'UTR fragment were inserted at the corresponding position if their minor allele frequency in relevant Caucasian populations was > = 0.03. Importantly, both alleles were considered in the pattern matching phase. Significant patterns derived from the human-specific mature miRNAs were used to identify candidate target islands. Mature miRNAs that yield the most favorable alignments in these islands were recorded.

DNA from subjects was extracted from blood samples or lymphoblastoid cell lines using the GenePure system (Gentra Systems). DNA samples were genotyped using Affymetrix 6.0 arrays at The Centre for Applied Genomics at the University of Toronto using standard procedures. The MirSNPs were genotyped by ligase detection reaction (LDR) and Luminex^TM^ 100 flow cytometry [37,38]. Reaction conditions were as previously described [37]. A list of SNPs genotyped, their locations, and the genotyping primer sequences for the MirSNPs are listed in S1 Table.

### Data cleaning and linkage disequilibrium analysis

Before data cleaning, the Affymetrix 6.0 array data had an average completion rate of 99.0% across all SNPs, genome-wide. Genotype cleaning of the 2,002 SNPs of interest was first conducted with PLINK v1.07[39] and included removing SNPS that had <98% completion rates (n = 226), were monomorphic (n = 227), or had >1% rate of Mendelian errors (n = 9). All SNPs had HWE p-values >0.001. Custom software was then used to identify and remove 111 genotypes causing Mendelian errors from the remaining 1,540 SNPs. At the end of this cleaning process the completion rate for the Affymetrix SNPs of interest was 99.7%. The five custom genotyped SNPs were subject to the same cleaning pipeline, with a resultant 98.8% completion rate.

SNPs were analyzed for evidence of linkage disequilibrium using the posterior probability of linkage (PPL) framework as previously described [27] and as implemented in the software package KELVIN v2.4.0 [40,41]. Marker allele frequencies were estimated using MENDEL (version 7.0)[42]. Since LD was being evaluated in a region of linkage, the PPLD|L (posterior probability of linkage disequilibrium given linkage) was calculated. The prior probability of linkage disequilibrium given linkage was set to the standard value of 2%. Values of the PPLD < 2% therefore represent evidence against LD in the presence of linkage, while values > 2% indicate evidence in favor of LD in the presence of linkage.

### Luciferase reporter assay

HT-1080 fibrosarcoma cells (ATCC) were cultured in Eagle's Minimum Essential Medium Alpha, supplemented with fetal bovine serum to a final concentration of 10% and 50 to 100 I.U./mL penicillin and 50 to 100 (μg/mL) streptomycin (Thermofisher). 96-well plates were seeded with HT-1080 fibrosarcoma cells (ATCC) 24 hours prior to transfection to achieve 80% confluence at the time of transfection.

Luciferase reporter constructs containing RenSP and the human 3′-untranslated region (3′UTRs) for *H3F3B* containing either the G (reference) or A (ancestral) allele of the SNP rs1060120, were transfected using the manufacturer’s protocol in conjunction with the Cypridina TK control construct driven by an HSV-TK constitutive promoter (SwitchGear Genomics). The transfection was performed using FuGENE HD* Transfection Reagent (Promega). The LightSwitch Dual Assay System (Switchgear Genomics) was used to normalize for variation between experimental replicates due to transfection variability by utilizing a co-transfection control.

Wells were co-transfected with either the miR-616-3p mimic (chemically optimized synthetic double-stranded RNAs that act as functional equivalents to endogenous human miRNAs; Catalog #MIM0611, Switchgear Genomics) or the non-targeting scramble miRNA (Non-targeting 1: Catalog #MIM9001, Switchgear Genomics) to yield a final concentration of 20 nm. Triplicate independent transfections were performed using a minimum of three replicate wells for each reaction experimental condition. The three independent transfections were performed consecutively over the course of several weeks.

Luciferase expression levels were measured using a Veritas Microplate luminometer (Turner Biosystems) following the protocol from the BioLux Cypridina Luciferase Assay (New England Biolabs).

The luciferase expression levels were normalized following manufacturer’s instructions for the Dual Luciferase Assay by dividing the LightSwitch Reagent values for each condition by the Cypridina co-transfection control values (Active Motif). To normalize the luciferase datacall values were divided by the luciferase measurement for the G UTR.

The data was analyzed in accordance with the randomized complete block design by using an additive term for trial in the ANOVA. Doing so accounts for trial-to-trial variability. Tukey's HSD (honestly significant difference) test was used to compare treatment differences; “adjusted p-values” in the text refer to the Tukey HSD adjustment. The R statistical environment was used for analysis.

## Results

### Predicted binding of miRNAs to SNP allele sites

In order to identify miRNAs that may be important for the development of schizophrenia, a custom miRNA target prediction program was used to identify SNPs under the linkage peak that were predicted to alter miRNA binding sites (MirSNPs). A total of five MirSNPs were predicted from the 17q25 region of this linkage peak, chr17: 74,684,647 to 83,257,441 (GRCh38): rs1060120 in gene *H3F3B*, rs4969391 in gene *BAIAP2*, rs7211218 in predicted gene *LOC283999*, rs1663196 in gene *TBC1D16*, and rs1128687 in gene *CHMP6* (Table 1).

**Table 1:** Predicted MirSNPs from the chromosome 17q25 region of association/linkage to broadly defined schizophrenia.

SNP name (“MirSNPs”)	17q25 region gene	dbSNP RefSNP alleles	miRNA(s) with binding site predicted to include SNP	SNP allele having stronger predicted binding to miRNAs
rs1060120	*H3F3B*	A/G	hsa-miR-616	Binding strength equal for both A and G alleles
rs4969391	*BAIAP2*	A/G	hsa-miR-1207-3p	A
rs7211218	*LOC283999*	A/C	hsa-miR-1245	C
rs1128687	*CHMP6*	C/T	hsa-miR-1827	C
rs1663196	*TBC1D16*	C/T	None^1^	-

^1^ Although SNP rs1663196 falls inside a "pattern island" (i.e., a region that has a high number of matches to the patterns extracted from the entire corpus of mature miRNAs in mirbase release 12.0), none of the known miRNAs yielded an alignment.

### Linkage disequilibrium analysis

**Fig 1:**
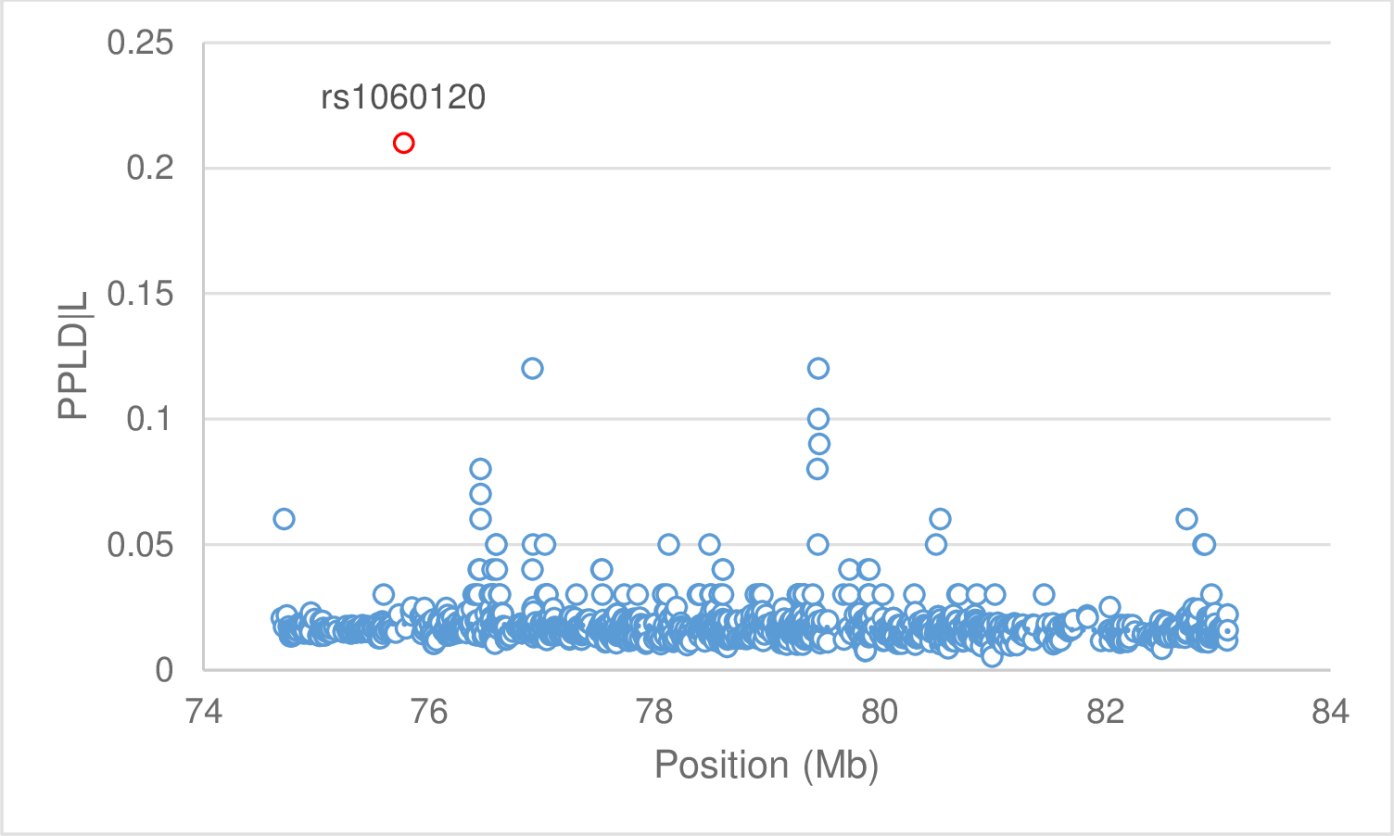
Linkage disequilibrium between 1,544 SNPs and broad schizophrenia spectrum phenotype. PPLD|L values for 1,544 SNPs, including five MirSNPs (Table 1), from chr17: 74,684,647 to 83,257,441 (GRCh38), were calculated using KELVIN v2.4.0 and plotted vs physical distance. The MirSNP rs1060120 in *H3F3B* produced a PPLD|L of 0.21, notably higher than the remaining SNPs.

Using the broad schizophrenia spectrum phenotype, PPLD|L values were calculated for 1,540 SNPs from the Affymetrix 6.0 Array and the five MirSNPs. One SNP (rs7211218) was on the array and was a predicted MirSNP, thus, in total 1,544 SNPs were tested. Overall, 1,338 SNPs (87%) of the 1,544 tested from the 17q25 region produced PPLD|L scores < 0.02, indicating evidence against LD. Only three SNPs produced PPLD|L scores > 0.10. SNP rs894941 and rs7220244 from the Affymetrix 6.0 Array each produced scores of 0.12; SNP rs1060120, the MirSNP in the 3’UTR of gene *H3F3B*, produced a score of 0.21. For rs1060120, the A allele was found to be associated with illness. Notably, rs1060120 was predicted to colocalize with potential miRNA target miR-616 (Table 1). All broad spectrum PPLD|L scores are plotted (Fig 1) and listed in S2 Table. We also analyzed rs1060120 for evidence of association to the narrow schizophrenia phenotype; for this phenotype the PPLD|L was only 0.06.

### Functional luciferase reporter assay

**Fig 2:**
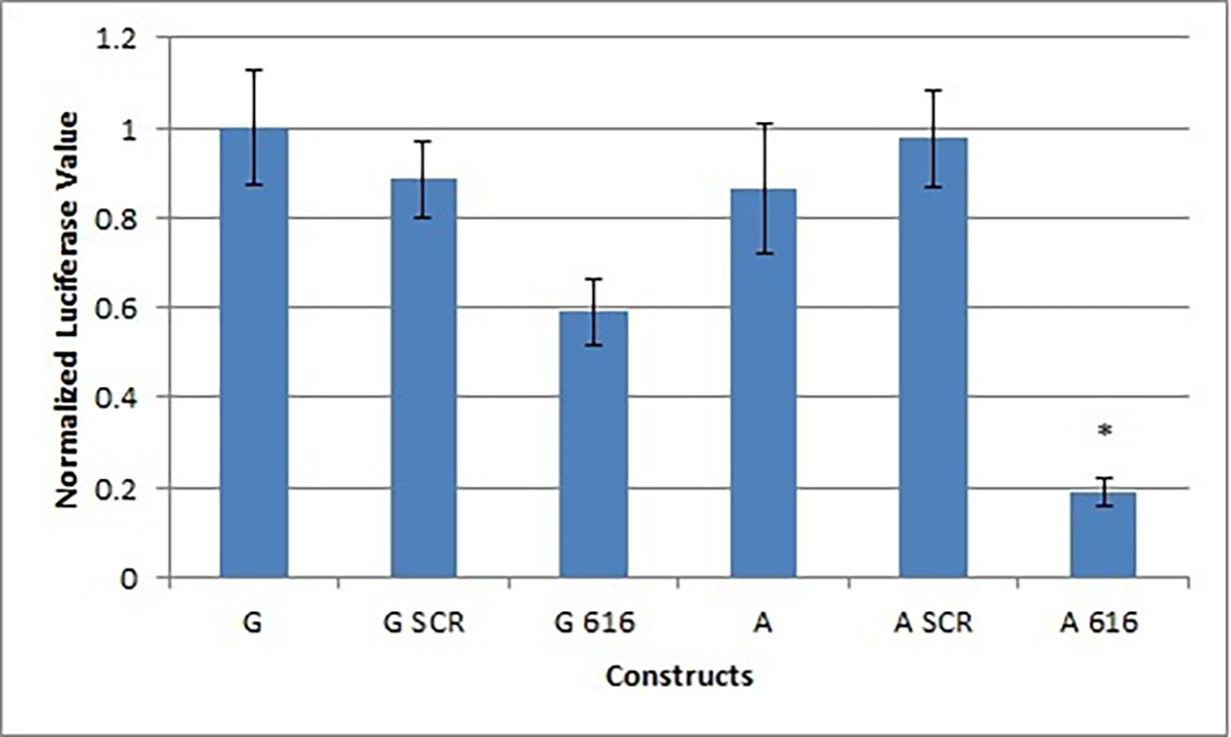
Dual luciferase reporter assay of broad schizophrenia-associated MirSNP rs1060120. The average normalized value for all three trials of the dual luciferase assay are plotted, with error bars representing the standard deviation for the A allele alone (A), the A Allele co-transfected with a nonspecific scramble miRNA mimic (A SCR), the A allele co-transfected with miR-616 (A 616), the G allele alone (G), the G allele co-transfected with a nonspecific scramble miRNA mimic (G SCR), and the G allele co-transfected with miR-616 (G 616). Data was analyzed with ANOVA using a term for the trial, in accordance with the randomized complete block design. There was evidence of a treatment effect (*p* < 1 x 10^−15^). Luciferase expression for the A construct co-transfected with miR-616 was significantly lower than for the G construct co-transfected with mir-616 (p-adjusted = 0.000412)(indicated by *). The nonspecific scramble miRNA mimic was not significantly different from the untreated constructs of either allele.

To functionally confirm this prediction and test whether SNP rs1060120 had an impact on miRNA binding, we used luciferase reporter constructs that contained the 3’UTR for *H3F3B* with either the A allele or the G allele. In the absence of added miRNA, expression of the two UTR variants was observed to be equivalent. Co-transfection of a scramble miRNA resulted in a slight decrease in expression, likely due to non-specific minor toxic effects of the construct. Co-transfection with the miR-616 mimic produced significantly decreased expression for both 3’ UTR variants (G-allele construct co-transfected with miR-616 mimic vs co-transfected with scramble: p adjusted = 0.0000336; A-allele construct co-transfected with miR-616 mimic vs co-transfected with scramble: p adjusted = < 1 x 10−7). The rs1060120 A-allele containing variant however exhibited a significantly greater decrease in luciferase expression (p adjusted = 0.000412) than the 3’UTR G-allele variant (Fig 2).

## Discussion

Prior PPL analysis of this set of families identified strong linkage peaks on chromosomes 1 and 17 [23]. We have previously characterized a functional SNP in gene NOS1AP under the chromosome 1 peak that exhibits strong linkage disequilibrium to a narrow schizophrenia phenotype in this sample [27]. We now provide evidence that in addition there is a functional SNP, rs1060120, in gene *H3F3B* located under the chromosome 17 peak that is in strong linkage disequilibrium with a broad schizophrenia spectrum phenotype in this sample.

Consistent with the genetic signal only emerging when considering a broad schizophrenia spectrum phenotype, our own sample shows limited support for association between the *H3F3B* SNP rs1060120 and a narrow definition of schizophrenia. Notably, a recent PGC study taking a broad diagnostic approach to pathway analysis of SNP data reported that the top pathway implicated genes involved in histone H3-K4 methylation[43], whereas GWAS using similar narrowly defined phenotypes, including the PGC [44], have not reported significant association to variants in *H3F3B*. It is possible that some proportion of controls in a case-control study could meet the criteria for broadly defined schizophrenia, thereby decreasing the signal to noise ratio. Also possible is that the relative genetic homogeneity of the familial schizophrenia sample used in this study has, by chance, provided a genetic background upon which the rs1060120 variant could exhibit a stronger effect than in a highly heterogeneous GWAS sample. Such background or strain effects have been well documented in genetic studies using animal models [45–48].

The associated SNP, rs1060120, is located in the 3’ UTR of gene *H3F3B*. Using a dual luciferase reporter assay in a mammalian cell culture system, we have validated a miR-616 target site in this 3’ UTR by showing that the schizophrenia spectrum-associated A allele results in greater miRNA-mediated reduction in luciferase than the G allele. The luciferase reporter tagged expression observed when the A allele construct was co-transfected with miR-616 was significantly greater than when the G allele construct was co-transfected with mir-616 (p adjusted = 0.000412) (Fig 2). These data suggest that allelic variants in rs1060120 could affect the function of miR-616 in individuals with a schizophrenia spectrum diagnosis, supporting the possibility that alteration of this miRNA/3’UTR interaction could be a factor contributing to expression of these disorders. Presence of the susceptibility allele could lead to decreased expression of *H3F3B*. This in turn could have an impact on the function of histones and their epigenetic modifications, key mechanisms previously implicated in the development of schizophrenia [49–51]. Such mechanisms in turn affect multiple genes active during brain development and later functioning, consistent with the mounting evidence for polygenic underpinning of schizophrenia[52–54].(52–54)

This miRNA mir-616/3’UTR *H3F3B* interaction has only been tested in cellular models. Therefore, ideally, one would determine if this interaction is indeed active in brain tissue during development and/or later and is altered in individuals who eventually express psychiatric illness. miRNAs have long been believed to play a role in the development of schizophrenia [55,56]. Further research on the relationship between this miRNA and its target 3’UTR would extend knowledge of miRNA targeting interactions. This could contribute to developing a greater understanding of the causes and complex mechanisms involved in the pathogenesis of schizophrenia and related disorders.

## Conclusion

Searching under a previously reported linkage peak on 17q25 [23], we have identified a functional SNP in the H3 histone gene *H3F3B* that was in strong linkage disequilibrium with a broad schizophrenia spectrum phenotype within the same sample that demonstrated linkage. The associated SNP, rs1060120, was found to be located within the 3’ UTR of *H3F3B*. The risk allele (A) was confirmed, using a dual luciferase reporter assay in a mammalian cell culture system, to functionally alter the targeting of miR-616. It is therefore possible this common variant could alter the function of miR-616 and thereby contribute to risk of schizophrenia spectrum diagnoses through downstream effects.

## Supporting information

S1 Table SNPs genotyped and the genotyping primer sequences for MirSNPs. (DOCX)

S2 Table All broad spectrum PPLD|L scores. (DOCX)
