# Supplementary material for: Validation of a microRNA target site polymorphism in H3F3B that is potentially associated with a broad schizophrenia phenotype
Source: PLoS One. 2018 Mar 12;13(3):e0194233. doi: 10.1371/journal.pone.0194233 (PMC5847241; doi:10.1371/journal.pone.0194233)
Supplement: S1 Table — (DOCX) [file pone.0194233.s001.docx]

**S1 Table: SNPs genotyped and the genotyping primer sequences for MirSNPs.**

| **SNPs from chr 17** | **Gene** | **Location** | **Forward Primer Sequence** | **Reverse Primer Sequence** | **Probe** | **Probe Sequence**  **(Tags in Bold)** |
| --- | --- | --- | --- | --- | --- | --- |
| rs1060120 | H3F3B | 17q25.1 | ATCACCGTAGCCAGGTTT | TCTCAAAAGTTTGGGTTAGTTTCA | rs1060120-C | **TACACATCTTACAAACTAATTTCA**AAGCGGGTCATTATATTGCATC |
|  |  |  |  |  | rs1060120-T | **ATACAATCTAACTTCACTATTACA**AAGCGGGTCATTATATTGCATT |
|  |  |  |  |  | rs1060120-com | CTTTCATAGGAGCTCATTATCACG**CTATCTTTAAACTACAAATCTAAC** |
| rs4969391 | BAIAP2 | 17q25.3 | CATCCAGCACTGGGGAAT | TGATGACAGAACAGGCTACA | rs4969391-C | **TACATACACTAATAACATACTCAT**ACTGTCGGCTTCAGCC |
|  |  |  |  |  | rs4969391-T | **TCATTTCAATCAATCATCAACAAT**ACTGTCGGCTTCAGCT |
|  |  |  |  |  | rs4969391-com | GGACGTGGGCAAAGGGC**TATCTTTAAACTACAAATCTAAC** |
| rs7211218 | LOC283999 | 17q25.3 | CCTCTTCCTCCTGGTGTTT | TGCTCACGTTTGTACTGTTT | rs7211218-A | **CAATTCATTTCATTCACAATCAAT**CATTGTGCCAATTTGTCCTTAA |
|  |  |  |  |  | rs7211218-C | **TTCACTTTTCAATCAACTTTAATC**CATTGTGCCAATTTGTCCTTAC |
|  |  |  |  |  | rs7211218-com | GTAAATCACTTGTGCTGTGTG**CTATCTTTAAACTACAAATCTAAC** |
| rs1663196 | TBC1D16 | 17q25.1963 | CAGTTCCCTTCGTGTACCA | TGGAAAGGCATCTGCCATA | rs1663196-C | **CTACAAACAAACAAACATTATCAA**AAGCCTGACTGCTCTTCC |
|  |  |  |  |  | rs1663196-T | **CTAACTAACAATAATCTAACTAAC**AAGCCTGACTGCTCTTCT |
|  |  |  |  |  | rs1663196-com | GCCTCTCTATCCCCACATC**CTATCTTTAAACTACAAATCTAAC** |
| rs1128687 | CHMP6 | 17q25.3 | TTCCGTTAAGGGGTCCCT | TCAAACTGGAGGTGGGTG | rs1128687-C | **TCATCAATCAATCTTTTTCACTTT**GCATGTGCTGAACCTCTCC |
|  |  |  |  |  | rs1128687-T | **TTACCTTTATACCTTTCTTTTTAC**GCATGTGCTGAACCTCTCT |
|  |  |  |  |  | rs1128687-com | GTGCCTCTGCCTCCG**CTATCTTTAAACTACAAATCTAAC** |
