# Supplementary material for: Validation of a microRNA target site polymorphism in H3F3B that is potentially associated with a broad schizophrenia phenotype
Source: PLoS One. 2018 Mar 12;13(3):e0194233. doi: 10.1371/journal.pone.0194233 (PMC5847241; doi:10.1371/journal.pone.0194233)
Supplement: S2 Table — (DOCX) [file pone.0194233.s002.docx]

**S2 Table: All broad spectrum PPLD|L scores.**

| **SNP** | **Position** | **PPLD\|L** |
| --- | --- | --- |
| rs11077753 | 74559871 | 0.0158 |
| rs1719478 | 74573180 | 0.0131 |
| rs1522874 | 74576619 | 0.0134 |
| rs783238 | 74590500 | 0.0129 |
| rs12453454 | 74596816 | 0.0164 |
| rs1027719 | 74601600 | 0.0133 |
| rs513643 | 74603279 | 0.015 |
| rs508255 | 74603865 | 0.0149 |
| rs482409 | 74604359 | 0.0149 |
| rs492256 | 74608037 | 0.0159 |
| rs563530 | 74608578 | 0.0162 |
| rs579238 | 74612695 | 0.0173 |
| rs509574 | 74612787 | 0.0162 |
| rs11077757 | 74614984 | 0.0165 |
| rs581157 | 74617450 | 0.021 |
| rs16978139 | 74621376 | 0.0172 |
| rs2455940 | 74622106 | 0.018 |
| rs1699607 | 74623086 | 0.0232 |
| rs480399 | 74625115 | 0.0226 |
| rs16978145 | 74626334 | 0.0172 |
| rs557064 | 74626464 | 0.0232 |
| rs556985 | 74626495 | 0.0226 |
| rs536263 | 74634292 | 0.0161 |
| rs11657185 | 74637676 | 0.0158 |
| rs8072111 | 74641126 | 0.0161 |
| rs7224452 | 74644553 | 0.0165 |
| rs7210053 | 74645052 | 0.0158 |
| rs477963 | 74645301 | 0.0172 |
| rs507765 | 74649473 | 0.0157 |
| rs496525 | 74649950 | 0.0153 |
| rs516209 | 74652863 | 0.0168 |
| rs513805 | 74652981 | 0.0159 |
| rs8081794 | 74657093 | 0.02 |
| rs7209136 | 74657903 | 0.0148 |
| rs2078152 | 74658847 | 0.0148 |
| rs1447344 | 74665139 | 0.0148 |
| rs7210132 | 74666932 | 0.0148 |
| rs11651871 | 74667428 | 0.0148 |
| rs11077758 | 74667628 | 0.0148 |
| rs9904078 | 74670881 | 0.0147 |
| rs16978165 | 74675481 | 0.0172 |
| rs2034310 | 74695789 | 0.0206 |
| rs5018105 | 74716223 | 0.06 |
| rs5018106 | 74716250 | 0.0171 |
| rs6501734 | 74739490 | 0.0216 |
| rs9904111 | 74755524 | 0.0172 |
| rs7420 | 74769076 | 0.0133 |
| rs734232 | 74769592 | 0.0152 |
| rs895691 | 74770161 | 0.0151 |
| rs880827 | 74771296 | 0.0135 |
| rs2305215 | 74772662 | 0.016 |
| rs9892802 | 74774158 | 0.016 |
| rs6501737 | 74784691 | 0.0133 |
| rs4789097 | 74785069 | 0.0137 |
| rs2384957 | 74788491 | 0.0136 |
| rs895690 | 74788510 | 0.0144 |
| rs3785533 | 74795932 | 0.0159 |
| rs872640 | 74797594 | 0.0157 |
| rs7226145 | 74804288 | 0.0155 |
| rs10512601 | 74805567 | 0.0157 |
| rs2385067 | 74813931 | 0.0143 |
| rs16978198 | 74815204 | 0.0159 |
| rs16978201 | 74833746 | 0.016 |
| rs3803783 | 74838446 | 0.0163 |
| rs1568447 | 74840873 | 0.0155 |
| rs7221436 | 74853558 | 0.0183 |
| rs690578 | 74857308 | 0.0171 |
| rs690307 | 74863587 | 0.0167 |
| rs560323 | 74868645 | 0.0185 |
| rs689639 | 74880025 | 0.0183 |
| rs689882 | 74880688 | 0.0159 |
| rs690616 | 74892235 | 0.0178 |
| rs690607 | 74892286 | 0.0173 |
| rs689992 | 74894376 | 0.0144 |
| rs2467578 | 74897639 | 0.0179 |
| rs689978 | 74898412 | 0.0174 |
| rs547011 | 74902396 | 0.0156 |
| rs571905 | 74909375 | 0.0188 |
| rs6501743 | 74939930 | 0.0171 |
| rs1542752 | 74942005 | 0.0175 |
| rs8073615 | 74944196 | 0.0148 |
| rs4789111 | 74944386 | 0.0175 |
| rs11649883 | 74948877 | 0.0143 |
| rs1994718 | 74949750 | 0.015 |
| rs1994720 | 74949983 | 0.0211 |
| rs4789114 | 74954832 | 0.0228 |
| rs10852758 | 74985680 | 0.0202 |
| rs1044228 | 75020526 | 0.0138 |
| rs4789126 | 75032295 | 0.0136 |
| rs10512598 | 75042037 | 0.0159 |
| rs9660 | 75042651 | 0.0194 |
| rs12938889 | 75043834 | 0.0161 |
| rs12103574 | 75056554 | 0.0195 |
| rs11077773 | 75063978 | 0.0167 |
| rs6501755 | 75074310 | 0.0137 |
| rs4789128 | 75074693 | 0.0137 |
| rs4789132 | 75082406 | 0.0154 |
| rs4788863 | 75093757 | 0.0158 |
| rs4238978 | 75100856 | 0.0154 |
| rs1531564 | 75114184 | 0.0159 |
| rs9891383 | 75116672 | 0.0147 |
| rs8082005 | 75117046 | 0.0167 |
| rs750844 | 75124833 | 0.016 |
| rs4789143 | 75132055 | 0.0148 |
| rs4789144 | 75132069 | 0.0165 |
| rs11656524 | 75160292 | 0.017 |
| rs729405 | 75189562 | 0.0156 |
| rs2242229 | 75246420 | 0.0156 |
| rs2242230 | 75246814 | 0.0151 |
| rs2242231 | 75246854 | 0.0151 |
| rs4789161 | 75266212 | 0.0174 |
| rs2385211 | 75284956 | 0.0157 |
| rs9944529 | 75285425 | 0.0155 |
| rs2306219 | 75286555 | 0.0153 |
| rs7217413 | 75300472 | 0.0153 |
| rs12603538 | 75301986 | 0.0153 |
| rs9892996 | 75307136 | 0.0145 |
| rs12936583 | 75308629 | 0.0148 |
| rs17490675 | 75317704 | 0.0177 |
| rs7219 | 75319287 | 0.0147 |
| rs16967789 | 75332908 | 0.0174 |
| rs11077787 | 75347263 | 0.0154 |
| rs9900586 | 75347846 | 0.0148 |
| rs1076094 | 75348630 | 0.0148 |
| rs4789175 | 75355739 | 0.0158 |
| rs8065193 | 75356475 | 0.0163 |
| rs2033607 | 75371207 | 0.0157 |
| rs4789182 | 75378864 | 0.0157 |
| rs6501786 | 75382694 | 0.0151 |
| rs4789188 | 75401790 | 0.0151 |
| rs4789189 | 75401904 | 0.0151 |
| rs930296 | 75408087 | 0.0152 |
| rs930297 | 75408456 | 0.0156 |
| rs4789193 | 75408715 | 0.0151 |
| rs16967866 | 75411300 | 0.0179 |
| rs7223674 | 75417368 | 0.0158 |
| rs8065160 | 75445009 | 0.0155 |
| rs6501798 | 75448566 | 0.0153 |
| rs6501800 | 75450810 | 0.0169 |
| rs6501801 | 75450949 | 0.0153 |
| rs6501803 | 75454144 | 0.0174 |
| rs6501805 | 75459847 | 0.0173 |
| rs7212620 | 75465849 | 0.0153 |
| rs8078577 | 75468213 | 0.0168 |
| rs3934780 | 75469235 | 0.0162 |
| rs4078474 | 75493486 | 0.0149 |
| rs8075850 | 75501358 | 0.0168 |
| rs7501644 | 75511785 | 0.0164 |
| rs4789205 | 75513460 | 0.0167 |
| rs6501824 | 75544532 | 0.0166 |
| rs6501825 | 75544774 | 0.0169 |
| rs12449601 | 75551694 | 0.0185 |
| rs11077793 | 75552782 | 0.0127 |
| rs1671035 | 75557847 | 0.0146 |
| rs960512 | 75565238 | 0.0128 |
| rs1661716 | 75571454 | 0.0132 |
| rs3862478 | 75572844 | 0.0127 |
| rs820240 | 75586946 | 0.0193 |
| rs2290454 | 75591333 | 0.0184 |
| rs736521 | 75592461 | 0.0171 |
| rs820255 | 75594441 | 0.0145 |
| rs820263 | 75598579 | 0.03 |
| rs1093992 | 75618277 | 0.0156 |
| rs1093994 | 75618714 | 0.0156 |
| rs820205 | 75644972 | 0.0161 |
| rs750339 | 75651286 | 0.0181 |
| rs820211 | 75654606 | 0.0161 |
| rs3785433 | 75670953 | 0.0182 |
| rs3785434 | 75671059 | 0.0178 |
| rs707707 | 75673441 | 0.0159 |
| rs820215 | 75697730 | 0.0152 |
| rs9897994 | 75708785 | 0.0149 |
| rs820172 | 75734402 | 0.022 |
| rs1060120 | 75776919 | 0.21 |
| rs17581498 | 75797966 | 0.0165 |
| rs8067076 | 75841061 | 0.0235 |
| rs936393 | 75851532 | 0.0217 |
| rs936391 | 75851718 | 0.0248 |
| rs7218738 | 75923457 | 0.0213 |
| rs2608881 | 75940572 | 0.0143 |
| rs3643 | 75941584 | 0.0218 |
| rs7217955 | 75950561 | 0.0218 |
| rs1135640 | 75953459 | 0.0166 |
| rs17583163 | 75961920 | 0.0193 |
| rs7226127 | 75963385 | 0.0246 |
| rs8065144 | 75963776 | 0.0157 |
| rs11651351 | 75970781 | 0.0172 |
| rs7342882 | 76007724 | 0.0185 |
| rs7342883 | 76007765 | 0.019 |
| rs2665993 | 76013130 | 0.0139 |
| rs2665964 | 76025223 | 0.0149 |
| rs17498898 | 76039363 | 0.0194 |
| rs16968440 | 76039610 | 0.02 |
| rs2305346 | 76040387 | 0.0114 |
| rs2260516 | 76040492 | 0.0136 |
| rs2598446 | 76041634 | 0.0104 |
| rs2598434 | 76049143 | 0.0105 |
| rs2665999 | 76060860 | 0.0125 |
| rs2443168 | 76070366 | 0.0118 |
| rs2290246 | 76087867 | 0.0197 |
| rs2665983 | 76090843 | 0.0199 |
| rs2457692 | 76096165 | 0.0192 |
| rs7501512 | 76097225 | 0.0185 |
| rs2666011 | 76099812 | 0.0185 |
| rs9895541 | 76101250 | 0.0187 |
| rs1868826 | 76131329 | 0.0154 |
| rs2598425 | 76146810 | 0.0157 |
| rs2598424 | 76152997 | 0.0159 |
| rs2585735 | 76153219 | 0.0157 |
| rs894544 | 76155375 | 0.0247 |
| rs4788915 | 76162720 | 0.0193 |
| rs9893558 | 76163682 | 0.0138 |
| rs16968642 | 76165452 | 0.0138 |
| rs1376356 | 76166425 | 0.0139 |
| rs11539879 | 76166467 | 0.0218 |
| rs8077046 | 76170788 | 0.0138 |
| rs10512608 | 76174909 | 0.0199 |
| rs9908949 | 76181305 | 0.0138 |
| rs4789251 | 76202737 | 0.0143 |
| rs12453112 | 76208276 | 0.0208 |
| rs12950642 | 76210259 | 0.0141 |
| rs12601803 | 76211803 | 0.0155 |
| rs9911122 | 76212343 | 0.0161 |
| rs6501862 | 76213651 | 0.0173 |
| rs10512605 | 76216301 | 0.0167 |
| rs8074389 | 76230379 | 0.0166 |
| rs3859181 | 76238577 | 0.0161 |
| rs7225367 | 76241453 | 0.0155 |
| rs8068689 | 76243540 | 0.0153 |
| rs8071545 | 76253925 | 0.0147 |
| rs9910028 | 76254129 | 0.0159 |
| rs8182357 | 76255988 | 0.0189 |
| rs4789264 | 76266967 | 0.0148 |
| rs347675 | 76279079 | 0.0176 |
| rs347674 | 76282467 | 0.015 |
| rs9905488 | 76287454 | 0.0163 |
| rs164009 | 76287588 | 0.0183 |
| rs346785 | 76287688 | 0.0183 |
| rs4789273 | 76290001 | 0.0171 |
| rs2279053 | 76291308 | 0.0162 |
| rs10459 | 76311656 | 0.0154 |
| rs447294 | 76312044 | 0.0208 |
| rs2279057 | 76313344 | 0.0193 |
| rs2279056 | 76313393 | 0.0193 |
| rs2279055 | 76313480 | 0.0173 |
| rs421578 | 76318692 | 0.0166 |
| rs402273 | 76337045 | 0.0147 |
| rs11077813 | 76340464 | 0.0232 |
| rs4789281 | 76362462 | 0.0184 |
| rs9909931 | 76372007 | 0.0179 |
| rs9910399 | 76372180 | 0.0179 |
| rs9889656 | 76372622 | 0.0175 |
| rs9892909 | 76376994 | 0.0163 |
| rs4789288 | 76377455 | 0.0166 |
| rs9989481 | 76378538 | 0.0187 |
| rs2958220 | 76379878 | 0.0242 |
| rs898085 | 76397600 | 0.03 |
| rs4789291 | 76410046 | 0.0139 |
| rs372126 | 76417134 | 0.03 |
| rs394873 | 76417367 | 0.0141 |
| rs7207108 | 76417803 | 0.0194 |
| rs443112 | 76417817 | 0.0192 |
| rs1533570 | 76425895 | 0.0138 |
| rs567009 | 76438735 | 0.0198 |
| rs1466003 | 76439277 | 0.03 |
| rs17596582 | 76442038 | 0.04 |
| rs4789297 | 76451269 | 0.04 |
| rs16968964 | 76459190 | 0.08 |
| rs11077821 | 76459497 | 0.07 |
| rs11077823 | 76459919 | 0.06 |
| rs8150 | 76470935 | 0.016 |
| rs3826287 | 76479963 | 0.0132 |
| rs8067774 | 76483807 | 0.014 |
| rs4788926 | 76491653 | 0.0139 |
| rs12948783 | 76503318 | 0.0223 |
| rs4648333 | 76518004 | 0.0174 |
| rs2289607 | 76540680 | 0.03 |
| rs9909792 | 76541492 | 0.0136 |
| rs895156 | 76545801 | 0.0238 |
| rs4648340 | 76546135 | 0.0244 |
| rs752049 | 76550857 | 0.0202 |
| rs7219690 | 76555886 | 0.0209 |
| rs16969025 | 76559141 | 0.0201 |
| rs3744051 | 76561928 | 0.0141 |
| rs3744052 | 76561975 | 0.0138 |
| rs4647887 | 76562724 | 0.0148 |
| rs7352 | 76565131 | 0.0142 |
| rs2241919 | 76565868 | 0.0143 |
| rs2166212 | 76566865 | 0.04 |
| rs2120886 | 76567093 | 0.0169 |
| rs2241918 | 76567571 | 0.03 |
| rs4789315 | 76570960 | 0.0146 |
| rs8076505 | 76572329 | 0.03 |
| rs11077829 | 76572425 | 0.03 |
| rs7209626 | 76578021 | 0.0154 |
| rs10512610 | 76578050 | 0.0152 |
| rs11077835 | 76582600 | 0.0164 |
| rs3809700 | 76587144 | 0.0105 |
| rs4788933 | 76597910 | 0.05 |
| rs4238998 | 76598042 | 0.05 |
| rs7214155 | 76600320 | 0.0177 |
| rs9904870 | 76602062 | 0.0174 |
| rs9898576 | 76605194 | 0.0183 |
| rs2590260 | 76605527 | 0.04 |
| rs11077846 | 76611665 | 0.0207 |
| rs11077847 | 76613603 | 0.024 |
| rs11656386 | 76618005 | 0.0186 |
| rs11650502 | 76618096 | 0.0164 |
| rs9903719 | 76619567 | 0.0182 |
| rs2410993 | 76622789 | 0.0227 |
| rs2286595 | 76625553 | 0.03 |
| rs12450896 | 76633785 | 0.03 |
| rs719430 | 76643448 | 0.0226 |
| rs973630 | 76645848 | 0.0181 |
| rs1117059 | 76646892 | 0.0177 |
| rs7223597 | 76651667 | 0.0173 |
| rs9889862 | 76651894 | 0.0226 |
| rs4261576 | 76652404 | 0.0224 |
| rs9915915 | 76656192 | 0.0177 |
| rs9913451 | 76656225 | 0.0175 |
| rs41380448 | 76657723 | 0.0225 |
| rs2410996 | 76657780 | 0.0225 |
| rs16969187 | 76660156 | 0.0175 |
| rs7221855 | 76668772 | 0.0154 |
| rs2159358 | 76675126 | 0.0171 |
| rs2286593 | 76685353 | 0.0171 |
| rs11077855 | 76685671 | 0.0122 |
| rs8075298 | 76685990 | 0.012 |
| rs8079290 | 76686119 | 0.012 |
| rs11077856 | 76692864 | 0.0123 |
| rs11868472 | 76705083 | 0.0139 |
| rs7215451 | 76705635 | 0.0137 |
| rs2240774 | 76714490 | 0.0133 |
| rs2286585 | 76716228 | 0.0135 |
| rs2240769 | 76722073 | 0.0134 |
| rs9916811 | 76753191 | 0.0152 |
| rs685441 | 76778743 | 0.0168 |
| rs9900513 | 76802292 | 0.0176 |
| rs7208733 | 76804917 | 0.0176 |
| rs1507072 | 76817065 | 0.0184 |
| rs1532200 | 76819524 | 0.0184 |
| rs2411036 | 76819603 | 0.0176 |
| rs8076858 | 76826599 | 0.0147 |
| rs12451489 | 76826911 | 0.0147 |
| rs12449967 | 76827199 | 0.0147 |
| rs397784 | 76828028 | 0.018 |
| rs12452461 | 76828627 | 0.0147 |
| rs7225404 | 76835725 | 0.016 |
| rs10048187 | 76843190 | 0.016 |
| rs17599976 | 76844492 | 0.0164 |
| rs16969378 | 76876496 | 0.0166 |
| rs11077873 | 76876823 | 0.0182 |
| rs9909585 | 76880812 | 0.016 |
| rs655149 | 76881391 | 0.0158 |
| rs546371 | 76882599 | 0.0149 |
| rs516063 | 76883635 | 0.0156 |
| rs557394 | 76889807 | 0.0158 |
| rs554885 | 76890055 | 0.0178 |
| rs4789363 | 76892597 | 0.0161 |
| rs4788951 | 76893064 | 0.0166 |
| rs4789364 | 76893183 | 0.0154 |
| rs7221665 | 76896149 | 0.0185 |
| rs532753 | 76896772 | 0.0163 |
| rs8064352 | 76900263 | 0.0172 |
| rs10852778 | 76902146 | 0.0153 |
| rs582096 | 76907695 | 0.0165 |
| rs1025934 | 76909513 | 0.0159 |
| rs12603564 | 76913017 | 0.0163 |
| rs2411042 | 76917855 | 0.0201 |
| rs736100 | 76918076 | 0.017 |
| rs16969506 | 76918859 | 0.0243 |
| rs12939833 | 76919014 | 0.0172 |
| rs894945 | 76919740 | 0.0155 |
| rs894944 | 76920005 | 0.04 |
| rs894941 | 76920422 | 0.12 |
| rs12936155 | 76921771 | 0.0176 |
| rs35640787 | 76922249 | 0.0188 |
| rs12451905 | 76924115 | 0.05 |
| rs7218723 | 76930346 | 0.025 |
| rs7220258 | 76930523 | 0.0232 |
| rs4789376 | 76933916 | 0.0147 |
| rs4789377 | 76934144 | 0.013 |
| rs4789378 | 76934284 | 0.0147 |
| rs9905472 | 76938454 | 0.0172 |
| rs8070977 | 76942889 | 0.0145 |
| rs920852 | 76942978 | 0.0156 |
| rs8074682 | 76944230 | 0.0146 |
| rs11869025 | 76951353 | 0.0189 |
| rs9807044 | 76975821 | 0.0136 |
| rs9807035 | 76975872 | 0.0149 |
| rs10512611 | 76989390 | 0.013 |
| rs7221059 | 76992362 | 0.0151 |
| rs11657575 | 76995442 | 0.0182 |
| rs2411032 | 76997524 | 0.0181 |
| rs8070840 | 77001992 | 0.0191 |
| rs3915813 | 77014411 | 0.0188 |
| rs11652334 | 77016994 | 0.0135 |
| rs9907918 | 77021323 | 0.0161 |
| rs755768 | 77027306 | 0.0196 |
| rs8070229 | 77029627 | 0.03 |
| rs12944373 | 77030208 | 0.0137 |
| rs8074577 | 77030770 | 0.05 |
| rs17609991 | 77032920 | 0.0135 |
| rs12150341 | 77045473 | 0.0179 |
| rs12951502 | 77045749 | 0.0153 |
| rs12938092 | 77048882 | 0.0119 |
| rs553157 | 77049144 | 0.016 |
| rs16969620 | 77053023 | 0.03 |
| rs9910269 | 77053563 | 0.03 |
| rs4628998 | 77063243 | 0.0153 |
| rs1124445 | 77064813 | 0.018 |
| rs9901870 | 77068055 | 0.0213 |
| rs10221221 | 77072313 | 0.0165 |
| rs751099 | 77074617 | 0.0202 |
| rs4789412 | 77075052 | 0.0162 |
| rs9915587 | 77075191 | 0.0187 |
| rs654505 | 77076596 | 0.0139 |
| rs544748 | 77076667 | 0.0214 |
| rs720825 | 77079582 | 0.0163 |
| rs716284 | 77079598 | 0.0139 |
| rs2018845 | 77079657 | 0.0214 |
| rs8069820 | 77081138 | 0.0157 |
| rs2678770 | 77089352 | 0.0146 |
| rs16969661 | 77089572 | 0.0167 |
| rs12600361 | 77091639 | 0.0163 |
| rs3817357 | 77098835 | 0.0181 |
| rs501316 | 77098889 | 0.0189 |
| rs597033 | 77100326 | 0.0148 |
| rs2585850 | 77102403 | 0.015 |
| rs689241 | 77106949 | 0.0244 |
| rs2053568 | 77113218 | 0.0205 |
| rs517459 | 77113952 | 0.015 |
| rs17533156 | 77118623 | 0.0171 |
| rs8079872 | 77119544 | 0.0142 |
| rs500416 | 77121770 | 0.0155 |
| rs652143 | 77126206 | 0.0145 |
| rs572850 | 77129191 | 0.0165 |
| rs569908 | 77129550 | 0.0168 |
| rs542539 | 77130188 | 0.015 |
| rs16969682 | 77130930 | 0.0162 |
| rs485500 | 77131840 | 0.0165 |
| rs16969683 | 77132113 | 0.0138 |
| rs12601162 | 77144416 | 0.0167 |
| rs2008417 | 77159694 | 0.0163 |
| rs2139461 | 77160323 | 0.0143 |
| rs12936917 | 77166882 | 0.0167 |
| rs7224114 | 77167932 | 0.0162 |
| rs11871056 | 77168804 | 0.0144 |
| rs11077892 | 77174591 | 0.0143 |
| rs7214763 | 77183805 | 0.0143 |
| rs8078846 | 77185016 | 0.0167 |
| rs12944803 | 77196071 | 0.0174 |
| rs2271792 | 77205447 | 0.0168 |
| rs2280271 | 77206305 | 0.0173 |
| rs1254795 | 77208466 | 0.0143 |
| rs8078674 | 77208805 | 0.0166 |
| rs17540750 | 77209063 | 0.0168 |
| rs7215278 | 77219021 | 0.0163 |
| rs606090 | 77221984 | 0.0159 |
| rs11077894 | 77231170 | 0.018 |
| rs17613220 | 77240868 | 0.0167 |
| rs11077897 | 77247861 | 0.0168 |
| rs1702925 | 77248934 | 0.0212 |
| rs678124 | 77249129 | 0.0162 |
| rs8065270 | 77250859 | 0.012 |
| rs2174919 | 77251197 | 0.0127 |
| rs10852781 | 77251875 | 0.0177 |
| rs2411107 | 77264304 | 0.0213 |
| rs9914452 | 77272529 | 0.0146 |
| rs16969881 | 77283643 | 0.0181 |
| rs1995755 | 77287459 | 0.016 |
| rs4788984 | 77292989 | 0.015 |
| rs8079187 | 77297220 | 0.0206 |
| rs6501948 | 77302905 | 0.0162 |
| rs2063197 | 77304319 | 0.0158 |
| rs7210195 | 77307792 | 0.0159 |
| rs4789440 | 77307814 | 0.0146 |
| rs11654929 | 77313269 | 0.03 |
| rs1436138 | 77320798 | 0.0138 |
| rs16969898 | 77323599 | 0.0173 |
| rs1347545 | 77324358 | 0.0149 |
| rs7502998 | 77336327 | 0.0135 |
| rs2411109 | 77336374 | 0.0142 |
| rs2060551 | 77342328 | 0.0152 |
| rs873370 | 77345322 | 0.0172 |
| rs387601 | 77350849 | 0.0126 |
| rs12450104 | 77351530 | 0.014 |
| rs11650316 | 77352089 | 0.012 |
| rs367539 | 77354790 | 0.0148 |
| rs8067472 | 77354895 | 0.012 |
| rs453370 | 77355341 | 0.0146 |
| rs7359497 | 77356091 | 0.0141 |
| rs182593 | 77357441 | 0.0139 |
| rs9907618 | 77363308 | 0.014 |
| rs312885 | 77365993 | 0.0155 |
| rs11650953 | 77371564 | 0.0155 |
| rs11077905 | 77372055 | 0.0147 |
| rs12451315 | 77376044 | 0.015 |
| rs178249 | 77385906 | 0.0193 |
| rs2002573 | 77386169 | 0.0144 |
| rs440375 | 77392118 | 0.0144 |
| rs391467 | 77392369 | 0.0144 |
| rs387774 | 77394822 | 0.0146 |
| rs403483 | 77395915 | 0.0146 |
| rs409785 | 77396170 | 0.0198 |
| rs406061 | 77398786 | 0.0163 |
| rs438163 | 77398895 | 0.016 |
| rs312864 | 77399361 | 0.016 |
| rs4789492 | 77401236 | 0.0164 |
| rs4789493 | 77401305 | 0.0161 |
| rs892961 | 77404018 | 0.0169 |
| rs1075457 | 77404655 | 0.0159 |
| rs9916143 | 77413200 | 0.0189 |
| rs11658267 | 77415802 | 0.0189 |
| rs11650011 | 77416252 | 0.0192 |
| rs8079522 | 77424154 | 0.02 |
| rs735397 | 77439424 | 0.0181 |
| rs367896 | 77486165 | 0.0148 |
| rs385419 | 77491120 | 0.0133 |
| rs312799 | 77493561 | 0.0131 |
| rs2574861 | 77511214 | 0.0154 |
| rs449988 | 77511597 | 0.0154 |
| rs312813 | 77512811 | 0.0172 |
| rs312812 | 77512855 | 0.0173 |
| rs6501958 | 77527497 | 0.0188 |
| rs12601604 | 77530918 | 0.0183 |
| rs920130 | 77531830 | 0.04 |
| rs2016792 | 77533232 | 0.0183 |
| rs41438052 | 77534881 | 0.0189 |
| rs12600934 | 77535078 | 0.0185 |
| rs4788990 | 77539015 | 0.04 |
| rs4471749 | 77540904 | 0.03 |
| rs8070652 | 77543537 | 0.0139 |
| rs11077912 | 77543689 | 0.0155 |
| rs8081210 | 77544463 | 0.0145 |
| rs11650965 | 77547288 | 0.0154 |
| rs7219060 | 77549430 | 0.0199 |
| rs7226077 | 77553656 | 0.017 |
| rs4789463 | 77554639 | 0.0162 |
| rs12601766 | 77554978 | 0.0162 |
| rs8072007 | 77566005 | 0.0109 |
| rs12602182 | 77568698 | 0.017 |
| rs4788996 | 77569622 | 0.0157 |
| rs8065893 | 77577974 | 0.0139 |
| rs2411130 | 77581221 | 0.0182 |
| rs2411131 | 77581295 | 0.0111 |
| rs11869200 | 77585598 | 0.0162 |
| rs9895691 | 77592727 | 0.0172 |
| rs11077917 | 77594500 | 0.015 |
| rs746300 | 77603078 | 0.0166 |
| rs1574511 | 77604896 | 0.0165 |
| rs16970334 | 77605290 | 0.0156 |
| rs9303008 | 77606041 | 0.0153 |
| rs4789473 | 77607126 | 0.0134 |
| rs2904494 | 77608099 | 0.017 |
| rs735342 | 77610610 | 0.0159 |
| rs3853833 | 77611154 | 0.0167 |
| rs1077758 | 77612590 | 0.0167 |
| rs16970364 | 77615853 | 0.019 |
| rs8077107 | 77616482 | 0.0156 |
| rs7350912 | 77626944 | 0.012 |
| rs9903049 | 77627252 | 0.0127 |
| rs9897125 | 77627536 | 0.013 |
| rs17546529 | 77628317 | 0.0135 |
| rs6501970 | 77632865 | 0.0159 |
| rs7501624 | 77635596 | 0.0157 |
| rs2077613 | 77643885 | 0.0115 |
| rs2411138 | 77644343 | 0.0152 |
| rs8075777 | 77644866 | 0.0213 |
| rs16970418 | 77645350 | 0.0202 |
| rs9907467 | 77646541 | 0.0154 |
| rs2870771 | 77650074 | 0.0122 |
| rs7503375 | 77650406 | 0.0121 |
| rs8066971 | 77652044 | 0.0122 |
| rs4427868 | 77652477 | 0.0122 |
| rs7218548 | 77658640 | 0.0141 |
| rs8070620 | 77661487 | 0.0136 |
| rs7220725 | 77662767 | 0.0145 |
| rs4789483 | 77662937 | 0.0173 |
| rs4789484 | 77664981 | 0.0106 |
| rs11652111 | 77671995 | 0.0224 |
| rs7207346 | 77672537 | 0.0138 |
| rs11869059 | 77672834 | 0.0223 |
| rs7221182 | 77679362 | 0.0135 |
| rs11077926 | 77686812 | 0.0162 |
| rs10451228 | 77688168 | 0.0148 |
| rs11077927 | 77690877 | 0.0141 |
| rs11651921 | 77693535 | 0.0148 |
| rs9891526 | 77699269 | 0.015 |
| rs9894103 | 77699327 | 0.015 |
| rs9901599 | 77700166 | 0.0145 |
| rs17548047 | 77700950 | 0.0138 |
| rs1550187 | 77704784 | 0.014 |
| rs9897643 | 77705494 | 0.0186 |
| rs9906155 | 77705615 | 0.0155 |
| rs17548096 | 77705700 | 0.0144 |
| rs10852786 | 77716844 | 0.0145 |
| rs9909030 | 77722470 | 0.0155 |
| rs934670 | 77722564 | 0.0139 |
| rs7213796 | 77723087 | 0.0157 |
| rs2033715 | 77723938 | 0.0157 |
| rs2033714 | 77723978 | 0.014 |
| rs16970482 | 77724221 | 0.0166 |
| rs16970485 | 77726350 | 0.0203 |
| rs9898235 | 77733406 | 0.03 |
| rs12601357 | 77734043 | 0.0196 |
| rs9889546 | 77739335 | 0.0137 |
| rs8080918 | 77742853 | 0.0167 |
| rs2118225 | 77745398 | 0.0137 |
| rs4449015 | 77745468 | 0.0129 |
| rs1865988 | 77748197 | 0.0151 |
| rs934668 | 77754686 | 0.0171 |
| rs875188 | 77755148 | 0.0139 |
| rs9907607 | 77755724 | 0.0121 |
| rs7214910 | 77764505 | 0.0117 |
| rs16970529 | 77765867 | 0.0138 |
| rs3906744 | 77768905 | 0.0189 |
| rs12947580 | 77772143 | 0.0145 |
| rs7208508 | 77778693 | 0.0116 |
| rs8075300 | 77785320 | 0.0134 |
| rs7224708 | 77786757 | 0.0196 |
| rs9909257 | 77789344 | 0.0177 |
| rs2033713 | 77798282 | 0.0169 |
| rs11649902 | 77799180 | 0.0155 |
| rs4319831 | 77808261 | 0.0177 |
| rs4523994 | 77808274 | 0.0119 |
| rs4319830 | 77808340 | 0.0152 |
| rs9914124 | 77813872 | 0.013 |
| rs9891854 | 77820926 | 0.0204 |
| rs4789502 | 77826130 | 0.0172 |
| rs12451966 | 77831289 | 0.0176 |
| rs9910961 | 77834041 | 0.0199 |
| rs4986072 | 77836798 | 0.0137 |
| rs4789504 | 77839563 | 0.0139 |
| rs4789505 | 77839886 | 0.0132 |
| rs4986069 | 77841795 | 0.0132 |
| rs8080480 | 77842944 | 0.0129 |
| rs11867731 | 77847892 | 0.0192 |
| rs9910318 | 77855384 | 0.03 |
| rs17550443 | 77874870 | 0.0208 |
| rs9893580 | 77875155 | 0.0124 |
| rs3935192 | 77882759 | 0.0182 |
| rs8066459 | 77905538 | 0.0137 |
| rs9897585 | 77914776 | 0.0126 |
| rs16970654 | 77916089 | 0.0179 |
| rs8081564 | 77924902 | 0.0121 |
| rs10512615 | 77926475 | 0.0105 |
| rs7212240 | 77928209 | 0.0172 |
| rs9896016 | 77942295 | 0.011 |
| rs17639052 | 78002155 | 0.0183 |
| rs9896371 | 78002289 | 0.0123 |
| rs17552162 | 78005696 | 0.0181 |
| rs4789523 | 78016773 | 0.0136 |
| rs17639398 | 78033188 | 0.0128 |
| rs4411562 | 78051531 | 0.0115 |
| rs16970774 | 78059466 | 0.0103 |
| rs16970784 | 78062601 | 0.0117 |
| rs4789012 | 78065485 | 0.0124 |
| rs4564643 | 78081813 | 0.03 |
| rs16970792 | 78087937 | 0.0148 |
| rs7221094 | 78089660 | 0.0178 |
| rs16970811 | 78095099 | 0.0234 |
| rs2311001 | 78096472 | 0.014 |
| rs2290907 | 78097596 | 0.0135 |
| rs2613513 | 78109650 | 0.0234 |
| rs2613514 | 78114437 | 0.03 |
| rs3794738 | 78123212 | 0.0167 |
| rs429216 | 78130123 | 0.05 |
| rs7208422 | 78134494 | 0.0116 |
| rs12452890 | 78134989 | 0.0191 |
| rs412611 | 78136178 | 0.0204 |
| rs8068430 | 78136611 | 0.015 |
| rs9906439 | 78151739 | 0.0179 |
| rs8073715 | 78155305 | 0.0182 |
| rs9909570 | 78162446 | 0.0143 |
| rs9897765 | 78172890 | 0.0112 |
| rs2661679 | 78176023 | 0.0212 |
| rs9893988 | 78176086 | 0.0121 |
| rs1811086 | 78176941 | 0.0221 |
| rs7217417 | 78177094 | 0.0212 |
| rs2854701 | 78177342 | 0.0228 |
| rs9903350 | 78186242 | 0.0214 |
| rs16970909 | 78194413 | 0.013 |
| rs8072874 | 78197826 | 0.025 |
| rs11655650 | 78221764 | 0.016 |
| rs12947167 | 78221902 | 0.0165 |
| rs2071214 | 78223510 | 0.018 |
| rs2661694 | 78224927 | 0.015 |
| rs1508146 | 78226482 | 0.0169 |
| rs1508147 | 78226507 | 0.016 |
| rs8078121 | 78235214 | 0.0188 |
| rs7211218 | 78240873 | 0.0172 |
| rs11658812 | 78241793 | 0.0166 |
| rs1567237 | 78250213 | 0.0127 |
| rs17641275 | 78251715 | 0.0136 |
| rs11657271 | 78253601 | 0.0131 |
| rs4789571 | 78279792 | 0.0127 |
| rs7217176 | 78280402 | 0.0164 |
| rs12938580 | 78291927 | 0.0125 |
| rs7220255 | 78293337 | 0.0118 |
| rs6501192 | 78294867 | 0.0099 |
| rs8071317 | 78305118 | 0.0148 |
| rs882358 | 78305795 | 0.0127 |
| rs9915291 | 78309532 | 0.0128 |
| rs4789580 | 78315910 | 0.0155 |
| rs9898312 | 78317528 | 0.0162 |
| rs4789579 | 78319350 | 0.0127 |
| rs7213427 | 78324048 | 0.0124 |
| rs4789576 | 78325905 | 0.0118 |
| rs4789575 | 78325952 | 0.0112 |
| rs8074003 | 78368412 | 0.0164 |
| rs2090205 | 78390193 | 0.03 |
| rs4969179 | 78395373 | 0.0159 |
| rs4969183 | 78397291 | 0.03 |
| rs16971105 | 78397894 | 0.0157 |
| rs16971107 | 78397961 | 0.0159 |
| rs2292641 | 78399553 | 0.0134 |
| rs12944051 | 78399775 | 0.0157 |
| rs4969186 | 78402323 | 0.0157 |
| rs938350 | 78403471 | 0.016 |
| rs2376583 | 78406024 | 0.03 |
| rs9900792 | 78411545 | 0.0178 |
| rs7225914 | 78412288 | 0.0168 |
| rs8071516 | 78420744 | 0.0168 |
| rs16971269 | 78435287 | 0.0154 |
| rs7211232 | 78446559 | 0.0174 |
| rs4969189 | 78448776 | 0.0114 |
| rs2289756 | 78460053 | 0.0163 |
| rs1465985 | 78474572 | 0.0176 |
| rs999430 | 78475597 | 0.02 |
| rs7405804 | 78476492 | 0.0169 |
| rs4969207 | 78476773 | 0.017 |
| rs8080976 | 78487814 | 0.0146 |
| rs11654661 | 78488866 | 0.0138 |
| rs11077372 | 78492202 | 0.05 |
| rs11077373 | 78493494 | 0.0146 |
| rs595711 | 78495227 | 0.0198 |
| rs17655474 | 78499404 | 0.0172 |
| rs691127 | 78501032 | 0.0175 |
| rs690844 | 78501838 | 0.03 |
| rs6501224 | 78506373 | 0.0153 |
| rs626439 | 78507542 | 0.03 |
| rs9902160 | 78508249 | 0.0233 |
| rs2120660 | 78508430 | 0.016 |
| rs2120661 | 78508455 | 0.019 |
| rs1515022 | 78509826 | 0.0188 |
| rs691043 | 78511630 | 0.0148 |
| rs11657650 | 78511884 | 0.0131 |
| rs7350961 | 78515861 | 0.0165 |
| rs6416834 | 78516079 | 0.013 |
| rs691336 | 78522344 | 0.0152 |
| rs642612 | 78525444 | 0.0126 |
| rs17729319 | 78525555 | 0.0169 |
| rs12453780 | 78525593 | 0.0156 |
| rs4273108 | 78529483 | 0.0122 |
| rs16971511 | 78533188 | 0.018 |
| rs2120658 | 78534211 | 0.0117 |
| rs7217596 | 78535782 | 0.0239 |
| rs7221209 | 78539866 | 0.0154 |
| rs7221694 | 78540130 | 0.0154 |
| rs4969166 | 78542766 | 0.0151 |
| rs4969167 | 78542822 | 0.0174 |
| rs16971523 | 78546535 | 0.0178 |
| rs4969206 | 78547439 | 0.0172 |
| rs8080186 | 78553070 | 0.0157 |
| rs11657098 | 78553817 | 0.0186 |
| rs17656528 | 78556601 | 0.0203 |
| rs11870238 | 78560062 | 0.0134 |
| rs16971539 | 78560534 | 0.0163 |
| rs1108366 | 78561861 | 0.0147 |
| rs1108367 | 78562691 | 0.0168 |
| rs9890885 | 78573730 | 0.0144 |
| rs1562102 | 78575478 | 0.013 |
| rs9904706 | 78575497 | 0.013 |
| rs2099971 | 78576688 | 0.0142 |
| rs12938153 | 78576847 | 0.0115 |
| rs12150121 | 78578193 | 0.0196 |
| rs16971566 | 78584294 | 0.03 |
| rs4573990 | 78585352 | 0.0146 |
| rs8067051 | 78585479 | 0.0147 |
| rs11651198 | 78598958 | 0.0159 |
| rs11651207 | 78599049 | 0.0147 |
| rs4969202 | 78601694 | 0.0198 |
| rs4969204 | 78601894 | 0.0109 |
| rs4969205 | 78602041 | 0.0104 |
| rs8072614 | 78602215 | 0.0112 |
| rs7220979 | 78608368 | 0.0221 |
| rs7220466 | 78608709 | 0.0141 |
| rs7406652 | 78609040 | 0.0225 |
| rs11077384 | 78609821 | 0.04 |
| rs9915834 | 78610659 | 0.03 |
| rs4796831 | 78611853 | 0.0202 |
| rs6501232 | 78612591 | 0.04 |
| rs9910295 | 78616363 | 0.0148 |
| rs9889754 | 78617066 | 0.0162 |
| rs4796808 | 78617593 | 0.0185 |
| rs4796795 | 78617767 | 0.0185 |
| rs4796796 | 78618038 | 0.0155 |
| rs11870479 | 78618496 | 0.0104 |
| rs4796798 | 78620465 | 0.0177 |
| rs4796799 | 78620845 | 0.0106 |
| rs7503527 | 78622344 | 0.0106 |
| rs4077596 | 78622614 | 0.0147 |
| rs7502312 | 78624851 | 0.0196 |
| rs7502404 | 78624946 | 0.0124 |
| rs12150681 | 78629364 | 0.0118 |
| rs4796803 | 78634082 | 0.0166 |
| rs9908993 | 78637503 | 0.0148 |
| rs9894631 | 78644609 | 0.0116 |
| rs9896611 | 78647879 | 0.0092 |
| rs11656024 | 78648485 | 0.0135 |
| rs12952382 | 78652629 | 0.012 |
| rs7207605 | 78653700 | 0.0142 |
| rs7209347 | 78654093 | 0.0124 |
| rs7212320 | 78654258 | 0.0142 |
| rs12948478 | 78658836 | 0.0197 |
| rs8076794 | 78665187 | 0.0126 |
| rs7215260 | 78681399 | 0.013 |
| rs12451377 | 78682128 | 0.0141 |
| rs9895721 | 78682858 | 0.0142 |
| rs9911523 | 78685744 | 0.0142 |
| rs10512617 | 78697469 | 0.0124 |
| rs11654527 | 78707434 | 0.0149 |
| rs7212406 | 78717841 | 0.0195 |
| rs8068876 | 78724727 | 0.0195 |
| rs17736494 | 78732586 | 0.0125 |
| rs9895025 | 78732638 | 0.017 |
| rs16971669 | 78736256 | 0.0195 |
| rs12943994 | 78741462 | 0.0195 |
| rs9903760 | 78741765 | 0.0127 |
| rs16971679 | 78744785 | 0.0195 |
| rs6501243 | 78748473 | 0.0129 |
| rs7224711 | 78776206 | 0.0127 |
| rs2889479 | 78788116 | 0.0126 |
| rs8182269 | 78793909 | 0.0125 |
| rs3744800 | 78797743 | 0.0131 |
| rs7211960 | 78808026 | 0.0145 |
| rs7212126 | 78808132 | 0.02 |
| rs12942645 | 78808234 | 0.0201 |
| rs2376828 | 78816758 | 0.0127 |
| rs7222131 | 78820117 | 0.0151 |
| rs3744793 | 78821008 | 0.0122 |
| rs1531797 | 78826086 | 0.0128 |
| rs11658299 | 78829640 | 0.0153 |
| rs9895419 | 78844832 | 0.0135 |
| rs1384364 | 78856746 | 0.0128 |
| rs16971783 | 78860571 | 0.0195 |
| rs11077399 | 78868093 | 0.0219 |
| rs2009196 | 78874499 | 0.0162 |
| rs9916773 | 78874536 | 0.0145 |
| rs1531796 | 78876298 | 0.0171 |
| rs4789940 | 78881589 | 0.0151 |
| rs3744787 | 78897589 | 0.0213 |
| rs12944916 | 78898183 | 0.019 |
| rs4789937 | 78898598 | 0.0195 |
| rs7211674 | 78902983 | 0.03 |
| rs12600817 | 78905383 | 0.0147 |
| rs6501265 | 78910003 | 0.0158 |
| rs8068674 | 78911363 | 0.0217 |
| rs4789934 | 78915454 | 0.0213 |
| rs12452379 | 78919376 | 0.0124 |
| rs9914634 | 78921031 | 0.0225 |
| rs8080623 | 78926196 | 0.0194 |
| rs9894526 | 78931004 | 0.0139 |
| rs4789855 | 78934960 | 0.0128 |
| rs7221260 | 78937563 | 0.03 |
| rs4789916 | 78952419 | 0.03 |
| rs7501823 | 78957097 | 0.0186 |
| rs11654023 | 78960186 | 0.0203 |
| rs8074370 | 78961249 | 0.0235 |
| rs11655528 | 78961717 | 0.03 |
| rs7222639 | 78962677 | 0.0233 |
| rs4789908 | 78965977 | 0.0179 |
| rs11871320 | 78968449 | 0.0115 |
| rs11077405 | 78973800 | 0.0177 |
| rs9789049 | 78980335 | 0.0168 |
| rs9914188 | 78981733 | 0.0188 |
| rs6501274 | 78981814 | 0.0142 |
| rs11891 | 78992703 | 0.0218 |
| rs3803781 | 78993700 | 0.0175 |
| rs2377301 | 78998287 | 0.0178 |
| rs17739056 | 78999123 | 0.0179 |
| rs12450407 | 79006681 | 0.0163 |
| rs4789903 | 79007245 | 0.0164 |
| rs11870326 | 79037010 | 0.0173 |
| rs4789912 | 79046240 | 0.0162 |
| rs4789911 | 79046266 | 0.0147 |
| rs3803780 | 79049340 | 0.019 |
| rs4789982 | 79085068 | 0.016 |
| rs10073 | 79088192 | 0.015 |
| rs7217829 | 79088593 | 0.0148 |
| rs7212486 | 79100095 | 0.0147 |
| rs2707051 | 79101004 | 0.0192 |
| rs753277 | 79108241 | 0.0175 |
| rs2612773 | 79119305 | 0.0132 |
| rs2703538 | 79119390 | 0.015 |
| rs2703537 | 79119655 | 0.0174 |
| rs2612793 | 79121499 | 0.0178 |
| rs11654190 | 79124045 | 0.0107 |
| rs9915970 | 79124117 | 0.0185 |
| rs12937891 | 79124495 | 0.0141 |
| rs7222155 | 79124589 | 0.0123 |
| rs12944841 | 79139479 | 0.0199 |
| rs4789947 | 79139709 | 0.0189 |
| rs1795952 | 79140596 | 0.0189 |
| rs7214618 | 79140715 | 0.0136 |
| rs2707040 | 79140752 | 0.0189 |
| rs1007252 | 79144117 | 0.0162 |
| rs7211029 | 79145226 | 0.0138 |
| rs7217802 | 79146309 | 0.0151 |
| rs4789949 | 79147378 | 0.0244 |
| rs1795961 | 79154698 | 0.0107 |
| rs2707030 | 79156760 | 0.0133 |
| rs881529 | 79158374 | 0.018 |
| rs2612782 | 79158854 | 0.0204 |
| rs9900690 | 79173272 | 0.0147 |
| rs8072165 | 79174496 | 0.0157 |
| rs8072667 | 79174518 | 0.0136 |
| rs11077415 | 79175673 | 0.0155 |
| rs2045660 | 79180269 | 0.0101 |
| rs8076283 | 79180711 | 0.0124 |
| rs7208724 | 79187239 | 0.0134 |
| rs11652768 | 79189868 | 0.014 |
| rs2703519 | 79190963 | 0.0152 |
| rs2612771 | 79191761 | 0.012 |
| rs2377309 | 79196998 | 0.0179 |
| rs12943811 | 79206717 | 0.0193 |
| rs16972153 | 79212099 | 0.0172 |
| rs2612753 | 79215972 | 0.0142 |
| rs9902768 | 79226508 | 0.0202 |
| rs7221761 | 79226597 | 0.0145 |
| rs4789960 | 79233380 | 0.0146 |
| rs7223911 | 79236004 | 0.0153 |
| rs9901008 | 79238441 | 0.0151 |
| rs1869934 | 79238589 | 0.0134 |
| rs12051618 | 79246981 | 0.018 |
| rs1378905 | 79256445 | 0.0123 |
| rs4789976 | 79260172 | 0.0145 |
| rs4789977 | 79262073 | 0.0188 |
| rs1006589 | 79267051 | 0.0145 |
| rs16972227 | 79267920 | 0.0193 |
| rs4789878 | 79270312 | 0.01 |
| rs4239026 | 79270927 | 0.0144 |
| rs11650709 | 79271487 | 0.0144 |
| rs898534 | 79277431 | 0.03 |
| rs897588 | 79277477 | 0.03 |
| rs897589 | 79277503 | 0.0133 |
| rs898533 | 79277537 | 0.03 |
| rs4789996 | 79287148 | 0.03 |
| rs9897367 | 79289286 | 0.03 |
| rs11656213 | 79289523 | 0.0207 |
| rs9898469 | 79289595 | 0.0132 |
| rs11656298 | 79289715 | 0.0176 |
| rs8082640 | 79299692 | 0.0228 |
| rs4789999 | 79300550 | 0.0124 |
| rs11655455 | 79302871 | 0.017 |
| rs12938262 | 79306006 | 0.0167 |
| rs4790005 | 79312700 | 0.0167 |
| rs8076416 | 79314371 | 0.0224 |
| rs4789885 | 79314413 | 0.022 |
| rs8078752 | 79315188 | 0.0186 |
| rs4790007 | 79317678 | 0.01 |
| rs12949118 | 79319266 | 0.0145 |
| rs12603040 | 79321443 | 0.016 |
| rs8065431 | 79322910 | 0.0161 |
| rs7219452 | 79325051 | 0.03 |
| rs9907544 | 79327552 | 0.0127 |
| rs11077428 | 79329244 | 0.0147 |
| rs12600765 | 79329641 | 0.0185 |
| rs9901936 | 79329838 | 0.0198 |
| rs8080101 | 79331014 | 0.03 |
| rs12946859 | 79331700 | 0.0142 |
| rs8064769 | 79334956 | 0.0165 |
| rs4592695 | 79335565 | 0.0165 |
| rs7212305 | 79337467 | 0.0165 |
| rs4790013 | 79338654 | 0.0125 |
| rs8076595 | 79338958 | 0.012 |
| rs4790032 | 79340656 | 0.0129 |
| rs12452661 | 79344278 | 0.0159 |
| rs12950551 | 79349606 | 0.0149 |
| rs907898 | 79349733 | 0.0165 |
| rs1877677 | 79350310 | 0.0142 |
| rs11870711 | 79351186 | 0.0167 |
| rs4790015 | 79353638 | 0.0165 |
| rs978415 | 79360612 | 0.0158 |
| rs12944016 | 79360933 | 0.0167 |
| rs11658442 | 79363311 | 0.0144 |
| rs11651690 | 79365496 | 0.0145 |
| rs11652791 | 79365779 | 0.0139 |
| rs8079646 | 79369452 | 0.0178 |
| rs2013350 | 79370707 | 0.0154 |
| rs12949424 | 79372071 | 0.0155 |
| rs2085351 | 79374564 | 0.0148 |
| rs4789892 | 79379569 | 0.0155 |
| rs7208287 | 79383025 | 0.0126 |
| rs925607 | 79385150 | 0.0165 |
| rs12451560 | 79388475 | 0.0129 |
| rs8070406 | 79389061 | 0.0236 |
| rs9911046 | 79391400 | 0.0182 |
| rs2124602 | 79392350 | 0.0199 |
| rs12938393 | 79392394 | 0.0168 |
| rs12601898 | 79393019 | 0.0131 |
| rs11656673 | 79404541 | 0.0172 |
| rs1106221 | 79407542 | 0.0239 |
| rs2377405 | 79408012 | 0.03 |
| rs872276 | 79415165 | 0.0156 |
| rs971626 | 79420858 | 0.0154 |
| rs10445220 | 79423194 | 0.0169 |
| rs6501303 | 79434511 | 0.0182 |
| rs8073156 | 79438384 | 0.0222 |
| rs8073024 | 79438402 | 0.015 |
| rs2137774 | 79440229 | 0.0146 |
| rs7210922 | 79444691 | 0.0166 |
| rs7207350 | 79446044 | 0.0153 |
| rs4476209 | 79446539 | 0.0153 |
| rs12451758 | 79450412 | 0.08 |
| rs11655935 | 79450788 | 0.0168 |
| rs4790044 | 79452549 | 0.0193 |
| rs11077442 | 79454625 | 0.05 |
| rs7209972 | 79458726 | 0.1 |
| rs7220244 | 79460227 | 0.12 |
| rs1028058 | 79468329 | 0.09 |
| rs6501311 | 79476792 | 0.0106 |
| rs4790047 | 79481744 | 0.0158 |
| rs4790051 | 79484243 | 0.0154 |
| rs296142 | 79487594 | 0.012 |
| rs296140 | 79487964 | 0.0143 |
| rs211788 | 79488175 | 0.012 |
| rs8070170 | 79492993 | 0.0124 |
| rs4790055 | 79493331 | 0.0189 |
| rs814747 | 79493863 | 0.0199 |
| rs7216806 | 79542435 | 0.011 |
| rs11871006 | 79545148 | 0.0197 |
| rs4074469 | 79680848 | 0.03 |
| rs4889908 | 79683660 | 0.0121 |
| rs8065292 | 79723327 | 0.0163 |
| rs4313838 | 79724812 | 0.0167 |
| rs4313839 | 79724849 | 0.0164 |
| rs4243252 | 79727803 | 0.0169 |
| rs8081537 | 79734466 | 0.03 |
| rs11657217 | 79735540 | 0.04 |
| rs8076446 | 79749785 | 0.0143 |
| rs9897213 | 79782565 | 0.0219 |
| rs4889787 | 79786965 | 0.0167 |
| rs745570 | 79807926 | 0.015 |
| rs7218526 | 79820061 | 0.0224 |
| rs8080723 | 79821213 | 0.0171 |
| rs7218751 | 79822638 | 0.0227 |
| rs1696757 | 79826944 | 0.0136 |
| rs1025925 | 79828320 | 0.0139 |
| rs1696743 | 79849064 | 0.0107 |
| rs894868 | 79851576 | 0.0185 |
| rs1696756 | 79854174 | 0.0168 |
| rs7208311 | 79854367 | 0.0151 |
| rs894864 | 79854991 | 0.0104 |
| rs894863 | 79855003 | 0.0104 |
| rs8072921 | 79859218 | 0.0115 |
| rs2650254 | 79859856 | 0.0198 |
| rs12936913 | 79860117 | 0.0109 |
| rs1285271 | 79860398 | 0.0198 |
| rs1696762 | 79865226 | 0.0091 |
| rs7406368 | 79872963 | 0.0096 |
| rs4889795 | 79873134 | 0.0098 |
| rs1285264 | 79875697 | 0.0075 |
| rs4243244 | 79877288 | 0.0192 |
| rs2587493 | 79878996 | 0.0076 |
| rs1285257 | 79890360 | 0.0195 |
| rs6565631 | 79893153 | 0.04 |
| rs11652759 | 79897012 | 0.0138 |
| rs12948255 | 79902563 | 0.03 |
| rs745603 | 79905143 | 0.03 |
| rs11656262 | 79907564 | 0.013 |
| rs1285285 | 79907910 | 0.0183 |
| rs1285286 | 79907978 | 0.0215 |
| rs1285288 | 79909545 | 0.04 |
| rs1285289 | 79909830 | 0.0149 |
| rs11655981 | 79917199 | 0.0205 |
| rs7220430 | 79917414 | 0.0133 |
| rs1620608 | 79917570 | 0.0137 |
| rs12940183 | 79917703 | 0.0141 |
| rs11658352 | 79918663 | 0.0154 |
| rs3169601 | 79935639 | 0.0153 |
| rs11150816 | 79936215 | 0.0143 |
| rs9799 | 79936357 | 0.0153 |
| rs935201 | 79937918 | 0.0152 |
| rs11150820 | 79938337 | 0.0144 |
| rs1663196 | 79940241 | 0.0168 |
| rs11150823 | 79945589 | 0.0136 |
| rs12451395 | 79955666 | 0.0229 |
| rs4889939 | 79964859 | 0.017 |
| rs4889810 | 80004440 | 0.0142 |
| rs894310 | 80030294 | 0.0151 |
| rs894309 | 80030415 | 0.03 |
| rs894308 | 80030491 | 0.0205 |
| rs11657363 | 80033814 | 0.0116 |
| rs11650121 | 80045981 | 0.0137 |
| rs2289529 | 80048446 | 0.0117 |
| rs2289531 | 80049923 | 0.0165 |
| rs9944501 | 80053096 | 0.0142 |
| rs9319623 | 80080807 | 0.014 |
| rs4889814 | 80084052 | 0.0137 |
| rs715041 | 80084633 | 0.0168 |
| rs1467979 | 80086944 | 0.0156 |
| rs1982243 | 80095298 | 0.0184 |
| rs1561810 | 80095525 | 0.014 |
| rs3816257 | 80100647 | 0.0183 |
| rs1442314 | 80103199 | 0.0154 |
| rs12600845 | 80103915 | 0.0173 |
| rs3816256 | 80105745 | 0.0178 |
| rs12452616 | 80116282 | 0.0156 |
| rs4889967 | 80116524 | 0.0156 |
| rs4889970 | 80122287 | 0.0158 |
| rs8064429 | 80122388 | 0.0175 |
| rs4889822 | 80124678 | 0.0205 |
| rs4889972 | 80124691 | 0.0205 |
| rs11150846 | 80124721 | 0.0147 |
| rs894315 | 80124901 | 0.0205 |
| rs12942941 | 80129214 | 0.0115 |
| rs2361719 | 80129604 | 0.0133 |
| rs12941119 | 80151658 | 0.0107 |
| rs12601192 | 80162268 | 0.0105 |
| rs8065486 | 80164397 | 0.0179 |
| rs12945249 | 80168501 | 0.0114 |
| rs12945399 | 80168729 | 0.0112 |
| rs12937978 | 80169642 | 0.0114 |
| rs12451566 | 80170232 | 0.0114 |
| rs4889989 | 80178061 | 0.0161 |
| rs7211994 | 80178715 | 0.0143 |
| rs2044102 | 80185561 | 0.0135 |
| rs2044103 | 80186413 | 0.0133 |
| rs3829612 | 80187603 | 0.0148 |
| rs3829611 | 80188016 | 0.0132 |
| rs8065364 | 80189160 | 0.0179 |
| rs8068452 | 80191712 | 0.0132 |
| rs2066964 | 80198145 | 0.0104 |
| rs2304856 | 80201684 | 0.0128 |
| rs2269376 | 80211728 | 0.0137 |
| rs2269375 | 80211757 | 0.0135 |
| rs6565651 | 80219453 | 0.0129 |
| rs12948768 | 80224006 | 0.0133 |
| rs6565653 | 80227984 | 0.0127 |
| rs7219513 | 80232700 | 0.0153 |
| rs6420487 | 80233891 | 0.0152 |
| rs11658788 | 80237933 | 0.0145 |
| rs6420489 | 80238407 | 0.0156 |
| rs8079641 | 80238621 | 0.0126 |
| rs8076666 | 80250018 | 0.0175 |
| rs9916351 | 80265733 | 0.0123 |
| rs12603583 | 80270438 | 0.0191 |
| rs9901491 | 80277719 | 0.0191 |
| rs8069705 | 80280268 | 0.0118 |
| rs12601730 | 80281798 | 0.0191 |
| rs9891691 | 80295200 | 0.0137 |
| rs9905727 | 80299014 | 0.0176 |
| rs8081176 | 80310187 | 0.03 |
| rs7501761 | 80316385 | 0.0229 |
| rs7222014 | 80319669 | 0.0101 |
| rs7501563 | 80326746 | 0.0129 |
| rs9913473 | 80327021 | 0.0129 |
| rs4075499 | 80327059 | 0.013 |
| rs9907978 | 80327580 | 0.0129 |
| rs9908583 | 80327674 | 0.0131 |
| rs8072917 | 80335308 | 0.0126 |
| rs6565677 | 80343642 | 0.0133 |
| rs9896984 | 80351468 | 0.019 |
| rs8078851 | 80352467 | 0.0129 |
| rs4078429 | 80361572 | 0.0167 |
| rs7221291 | 80370543 | 0.0135 |
| rs4889848 | 80380861 | 0.0171 |
| rs4890018 | 80386201 | 0.0197 |
| rs7223701 | 80388851 | 0.0166 |
| rs8359 | 80394251 | 0.0179 |
| rs4310923 | 80413707 | 0.0162 |
| rs11657930 | 80418434 | 0.0176 |
| rs8067574 | 80418591 | 0.0155 |
| rs9910924 | 80432236 | 0.0161 |
| rs4491586 | 80434191 | 0.0157 |
| rs4494603 | 80434413 | 0.017 |
| rs4318264 | 80439629 | 0.0158 |
| rs9901699 | 80453610 | 0.0175 |
| rs7211327 | 80453940 | 0.0112 |
| rs8068613 | 80457733 | 0.0151 |
| rs9898443 | 80495557 | 0.0144 |
| rs4561525 | 80502268 | 0.05 |
| rs7212223 | 80509760 | 0.0182 |
| rs12949799 | 80510627 | 0.0146 |
| rs9892081 | 80510684 | 0.0138 |
| rs7208637 | 80520355 | 0.0214 |
| rs12945047 | 80521169 | 0.0147 |
| rs11150860 | 80525021 | 0.0214 |
| rs4075979 | 80528608 | 0.0196 |
| rs7222801 | 80539301 | 0.02 |
| rs4890037 | 80539416 | 0.06 |
| rs3923310 | 80540653 | 0.0103 |
| rs3923514 | 80541410 | 0.0144 |
| rs9916371 | 80541727 | 0.0124 |
| rs7224941 | 80543108 | 0.0153 |
| rs11658698 | 80544527 | 0.0119 |
| rs5003528 | 80550330 | 0.0153 |
| rs4889857 | 80552698 | 0.0153 |
| rs4890043 | 80553031 | 0.0144 |
| rs11651818 | 80556500 | 0.0144 |
| rs12451038 | 80558425 | 0.0181 |
| rs11150864 | 80558951 | 0.0153 |
| rs4278795 | 80559389 | 0.0144 |
| rs12951500 | 80562925 | 0.0146 |
| rs4890047 | 80575639 | 0.0123 |
| rs4396581 | 80575934 | 0.0108 |
| rs11150866 | 80576213 | 0.0131 |
| rs4889863 | 80576668 | 0.0123 |
| rs7225476 | 80587803 | 0.0149 |
| rs12450647 | 80597242 | 0.0133 |
| rs9913181 | 80600072 | 0.0139 |
| rs7214653 | 80607650 | 0.0192 |
| rs1125587 | 80607896 | 0.0193 |
| rs8080957 | 80608893 | 0.0086 |
| rs12942637 | 80610071 | 0.0135 |
| rs11653499 | 80610503 | 0.0131 |
| rs12939613 | 80614402 | 0.0187 |
| rs12951779 | 80614926 | 0.0142 |
| rs9890502 | 80617225 | 0.0131 |
| rs7503807 | 80617311 | 0.0187 |
| rs4890056 | 80617411 | 0.0187 |
| rs11658093 | 80620734 | 0.013 |
| rs8075393 | 80620954 | 0.0187 |
| rs901063 | 80621896 | 0.0206 |
| rs901064 | 80622240 | 0.0193 |
| rs12952285 | 80622358 | 0.0152 |
| rs8080265 | 80631014 | 0.0131 |
| rs999977 | 80632612 | 0.0181 |
| rs2171023 | 80635113 | 0.0206 |
| rs1126064 | 80636588 | 0.0198 |
| rs1032130 | 80636836 | 0.0191 |
| rs12939549 | 80637924 | 0.0193 |
| rs11150736 | 80639707 | 0.0152 |
| rs8072592 | 80641561 | 0.0128 |
| rs12946972 | 80645122 | 0.0128 |
| rs12936687 | 80645209 | 0.0115 |
| rs12947901 | 80645442 | 0.0136 |
| rs7212125 | 80655063 | 0.0194 |
| rs9897453 | 80660500 | 0.0219 |
| rs11150737 | 80667490 | 0.0114 |
| rs6565472 | 80668820 | 0.0147 |
| rs7210015 | 80682687 | 0.0156 |
| rs901066 | 80685817 | 0.0194 |
| rs11652856 | 80687840 | 0.0156 |
| rs7501674 | 80688377 | 0.0194 |
| rs4889879 | 80688773 | 0.0189 |
| rs756077 | 80689271 | 0.0193 |
| rs9907193 | 80691399 | 0.03 |
| rs4889784 | 80694063 | 0.015 |
| rs884204 | 80695448 | 0.0133 |
| rs7208502 | 80696528 | 0.0156 |
| rs9911171 | 80697103 | 0.0161 |
| rs2315924 | 80704337 | 0.03 |
| rs8065598 | 80704443 | 0.0161 |
| rs11150739 | 80705179 | 0.0165 |
| rs8072229 | 80726019 | 0.014 |
| rs9896771 | 80730099 | 0.0126 |
| rs2315921 | 80732717 | 0.0122 |
| rs9915378 | 80734618 | 0.0135 |
| rs6565479 | 80738623 | 0.0128 |
| rs12941240 | 80743004 | 0.0114 |
| rs7213696 | 80747371 | 0.0115 |
| rs4602089 | 80750185 | 0.0139 |
| rs12601089 | 80751670 | 0.0139 |
| rs9902224 | 80751824 | 0.0185 |
| rs9899782 | 80751840 | 0.0185 |
| rs4396582 | 80751891 | 0.0185 |
| rs9902639 | 80751966 | 0.0185 |
| rs4969230 | 80752461 | 0.0133 |
| rs7215564 | 80763487 | 0.0195 |
| rs9913009 | 80763550 | 0.0173 |
| rs9891139 | 80768654 | 0.0175 |
| rs4969425 | 80787102 | 0.0142 |
| rs4969429 | 80791419 | 0.0203 |
| rs2589138 | 80795800 | 0.0161 |
| rs7219896 | 80801771 | 0.0152 |
| rs7214338 | 80802015 | 0.0153 |
| rs7214655 | 80802170 | 0.0153 |
| rs9908495 | 80804892 | 0.0153 |
| rs4969443 | 80806005 | 0.0153 |
| rs4969277 | 80806181 | 0.0153 |
| rs4969444 | 80806210 | 0.0153 |
| rs4969445 | 80806280 | 0.0153 |
| rs2672887 | 80808004 | 0.0143 |
| rs734338 | 80808540 | 0.0135 |
| rs9915426 | 80810512 | 0.0134 |
| rs8065459 | 80813189 | 0.0149 |
| rs2589133 | 80819676 | 0.0119 |
| rs9908195 | 80821755 | 0.0165 |
| rs9911574 | 80822068 | 0.0116 |
| rs7216306 | 80829775 | 0.0134 |
| rs2672871 | 80832513 | 0.0115 |
| rs7219745 | 80843108 | 0.0171 |
| rs2672900 | 80844567 | 0.0112 |
| rs2589155 | 80848417 | 0.0218 |
| rs2589153 | 80848789 | 0.0199 |
| rs908241 | 80861878 | 0.0196 |
| rs11150751 | 80865234 | 0.0191 |
| rs12939768 | 80865402 | 0.03 |
| rs8070964 | 80865426 | 0.0196 |
| rs8076420 | 80865578 | 0.0187 |
| rs2289759 | 80880423 | 0.016 |
| rs2589122 | 80881291 | 0.0174 |
| rs4969287 | 80890229 | 0.0137 |
| rs11656061 | 80890949 | 0.0136 |
| rs12232521 | 80899153 | 0.0171 |
| rs7217623 | 80899214 | 0.0156 |
| rs868432 | 80903935 | 0.0095 |
| rs1468030 | 80909040 | 0.0151 |
| rs7220598 | 80911890 | 0.0172 |
| rs7220348 | 80912058 | 0.0149 |
| rs7225616 | 80912982 | 0.0144 |
| rs9893657 | 80920521 | 0.0134 |
| rs9897968 | 80920714 | 0.0137 |
| rs2271607 | 80923346 | 0.014 |
| rs2271608 | 80925658 | 0.0135 |
| rs4969310 | 80928180 | 0.0169 |
| rs4969311 | 80932560 | 0.017 |
| rs9902338 | 80933509 | 0.0134 |
| rs11655742 | 80936261 | 0.018 |
| rs11653325 | 80936829 | 0.0171 |
| rs6565499 | 80939266 | 0.0167 |
| rs9915393 | 80942028 | 0.014 |
| rs6565500 | 80942689 | 0.0136 |
| rs8074171 | 80951849 | 0.0167 |
| rs7224748 | 80955220 | 0.015 |
| rs9899178 | 80961397 | 0.016 |
| rs11653897 | 80977377 | 0.0143 |
| rs1399572 | 80978587 | 0.0186 |
| rs11150760 | 80989081 | 0.0141 |
| rs4969325 | 80989132 | 0.0111 |
| rs4969326 | 80989145 | 0.0109 |
| rs4969331 | 80993115 | 0.0076 |
| rs1128687 | 80999674 | 0.0053 |
| rs8078438 | 81002795 | 0.0177 |
| rs8072124 | 81027500 | 0.03 |
| rs4969239 | 81036744 | 0.0139 |
| rs6565532 | 81049653 | 0.0126 |
| rs8067235 | 81050837 | 0.0169 |
| rs8069327 | 81051115 | 0.0152 |
| rs9914904 | 81051263 | 0.0188 |
| rs8080525 | 81055216 | 0.016 |
| rs8070741 | 81069175 | 0.0128 |
| rs4076037 | 81070484 | 0.0151 |
| rs8072172 | 81073313 | 0.0174 |
| rs4969381 | 81080876 | 0.0118 |
| rs8066330 | 81084478 | 0.0122 |
| rs8080815 | 81085430 | 0.0122 |
| rs7210438 | 81086380 | 0.0146 |
| rs4969385 | 81095090 | 0.0151 |
| rs4075483 | 81101017 | 0.0102 |
| rs11664 | 81109062 | 0.0133 |
| rs4969391 | 81115790 | 0.0178 |
| rs4969398 | 81136109 | 0.0118 |
| rs7211267 | 81139363 | 0.0171 |
| rs12449859 | 81158113 | 0.0161 |
| rs2659042 | 81161357 | 0.0096 |
| rs9911980 | 81168719 | 0.0136 |
| rs12103867 | 81176854 | 0.0141 |
| rs7209980 | 81181217 | 0.0182 |
| rs2659015 | 81199384 | 0.0186 |
| rs2725393 | 81211352 | 0.0177 |
| rs9893755 | 81213632 | 0.01 |
| rs2659029 | 81216762 | 0.0179 |
| rs9896850 | 81217926 | 0.0098 |
| rs9902277 | 81250047 | 0.0147 |
| rs7223939 | 81267721 | 0.0149 |
| rs11150779 | 81271103 | 0.014 |
| rs2271090 | 81272805 | 0.0143 |
| rs7216728 | 81273998 | 0.018 |
| rs7219316 | 81274520 | 0.0143 |
| rs11652842 | 81279778 | 0.0147 |
| rs10871494 | 81282198 | 0.0153 |
| rs10445407 | 81288009 | 0.0135 |
| rs12103824 | 81288651 | 0.0156 |
| rs8076302 | 81291901 | 0.0155 |
| rs12943170 | 81310502 | 0.0149 |
| rs9896868 | 81364197 | 0.018 |
| rs11652437 | 81364669 | 0.0119 |
| rs8073897 | 81375200 | 0.0179 |
| rs2306213 | 81462202 | 0.0139 |
| rs9902119 | 81462332 | 0.03 |
| rs7209156 | 81479566 | 0.016 |
| rs9907460 | 81485136 | 0.0183 |
| rs11871546 | 81494370 | 0.013 |
| rs7502122 | 81494460 | 0.013 |
| rs11654087 | 81538163 | 0.0172 |
| rs7406710 | 81538598 | 0.0186 |
| rs7406506 | 81543873 | 0.0103 |
| rs9911739 | 81565083 | 0.0124 |
| rs9911460 | 81571815 | 0.0109 |
| rs7405450 | 81573423 | 0.0141 |
| rs9911383 | 81574090 | 0.0158 |
| rs1122424 | 81593468 | 0.0172 |
| rs9913546 | 81604365 | 0.0137 |
| rs11652797 | 81607098 | 0.0118 |
| rs7222241 | 81607688 | 0.0118 |
| rs6565612 | 81635794 | 0.0181 |
| rs9905639 | 81647128 | 0.0172 |
| rs9906358 | 81647455 | 0.0165 |
| rs7406828 | 81652196 | 0.0172 |
| rs7502869 | 81664386 | 0.0174 |
| rs7209877 | 81670607 | 0.0161 |
| rs6565619 | 81678866 | 0.0154 |
| rs6565620 | 81691070 | 0.0159 |
| rs11867462 | 81708838 | 0.0161 |
| rs11868024 | 81709136 | 0.0161 |
| rs3204270 | 81715021 | 0.0166 |
| rs12449687 | 81726106 | 0.0198 |
| rs1130674 | 81845225 | 0.0217 |
| rs2070871 | 81847258 | 0.0208 |
| rs4239275 | 81965842 | 0.0115 |
| rs9903371 | 82035188 | 0.0156 |
| rs12936536 | 82046174 | 0.0116 |
| rs6502048 | 82047052 | 0.0159 |
| rs6502049 | 82047156 | 0.025 |
| rs8068796 | 82064485 | 0.0151 |
| rs6502050 | 82100360 | 0.0163 |
| rs6502051 | 82101456 | 0.0129 |
| rs7225637 | 82101882 | 0.0141 |
| rs7226049 | 82113173 | 0.0129 |
| rs11867806 | 82114173 | 0.0131 |
| rs11654584 | 82121559 | 0.0117 |
| rs4459614 | 82128721 | 0.0114 |
| rs4789754 | 82134065 | 0.0114 |
| rs4789753 | 82134255 | 0.0178 |
| rs8067875 | 82134902 | 0.0114 |
| rs7502676 | 82135557 | 0.0108 |
| rs9674648 | 82138880 | 0.0114 |
| rs3088177 | 82153848 | 0.0116 |
| rs3935402 | 82156376 | 0.0178 |
| rs9911379 | 82156653 | 0.0114 |
| rs8077772 | 82159344 | 0.0115 |
| rs8071425 | 82159459 | 0.0114 |
| rs9916231 | 82161769 | 0.0113 |
| rs4356529 | 82199921 | 0.0112 |
| rs8079572 | 82200976 | 0.0178 |
| rs4789667 | 82204267 | 0.0114 |
| rs11652189 | 82205012 | 0.0178 |
| rs8077209 | 82205345 | 0.013 |
| rs9747201 | 82219976 | 0.0126 |
| rs4510078 | 82247837 | 0.0178 |
| rs4789846 | 82267669 | 0.0153 |
| rs12450996 | 82336233 | 0.0138 |
| rs9944528 | 82342874 | 0.0144 |
| rs8072762 | 82343372 | 0.0145 |
| rs7207095 | 82381853 | 0.0136 |
| rs7211306 | 82409477 | 0.0124 |
| rs6502108 | 82417023 | 0.0129 |
| rs7217570 | 82421456 | 0.0132 |
| rs4789777 | 82436654 | 0.0135 |
| rs9648 | 82442710 | 0.0118 |
| rs8008 | 82442879 | 0.0154 |
| rs11077994 | 82446450 | 0.0112 |
| rs7209788 | 82458038 | 0.0128 |
| rs12952655 | 82463263 | 0.0139 |
| rs4789693 | 82463994 | 0.0139 |
| rs7406694 | 82469464 | 0.0126 |
| rs4789694 | 82470223 | 0.0139 |
| rs9909476 | 82481857 | 0.0155 |
| rs9898723 | 82482031 | 0.0149 |
| rs8081734 | 82490657 | 0.0125 |
| rs6502114 | 82491800 | 0.0123 |
| rs1317685 | 82491821 | 0.0172 |
| rs9897468 | 82492261 | 0.0197 |
| rs9901463 | 82506738 | 0.0084 |
| rs9911520 | 82506829 | 0.0166 |
| rs9898429 | 82522776 | 0.0138 |
| rs4789696 | 82523905 | 0.0155 |
| rs11077998 | 82526070 | 0.0154 |
| rs12450641 | 82531415 | 0.0137 |
| rs4789782 | 82531447 | 0.0153 |
| rs9904946 | 82536367 | 0.0184 |
| rs7219048 | 82544260 | 0.0189 |
| rs4789786 | 82546476 | 0.0153 |
| rs4614761 | 82547933 | 0.0153 |
| rs10852797 | 82551800 | 0.013 |
| rs4789788 | 82552409 | 0.0153 |
| rs11653963 | 82552506 | 0.0181 |
| rs4789789 | 82552835 | 0.0143 |
| rs4789795 | 82563216 | 0.0142 |
| rs12453001 | 82577265 | 0.013 |
| rs3794716 | 82601291 | 0.0171 |
| rs4822 | 82602618 | 0.0154 |
| rs4789704 | 82613184 | 0.0132 |
| rs2291392 | 82627157 | 0.0167 |
| rs11649788 | 82644174 | 0.0161 |
| rs9303031 | 82644721 | 0.0162 |
| rs4789817 | 82650113 | 0.0135 |
| rs8068482 | 82650273 | 0.015 |
| rs7226089 | 82652315 | 0.0134 |
| rs6502127 | 82653129 | 0.0164 |
| rs12952494 | 82659140 | 0.0136 |
| rs7210081 | 82662641 | 0.013 |
| rs9893830 | 82673645 | 0.0137 |
| rs11654159 | 82695073 | 0.0148 |
| rs3898431 | 82700463 | 0.0146 |
| rs12943478 | 82713475 | 0.0128 |
| rs2243523 | 82722573 | 0.0172 |
| rs2246563 | 82723196 | 0.0145 |
| rs4510058 | 82725842 | 0.0208 |
| rs1046889 | 82727635 | 0.06 |
| rs1046896 | 82727657 | 0.0151 |
| rs2264574 | 82745504 | 0.0216 |
| rs2451215 | 82757023 | 0.0176 |
| rs2451214 | 82757178 | 0.0199 |
| rs11868704 | 82768384 | 0.0196 |
| rs8066848 | 82786497 | 0.0245 |
| rs11650019 | 82792814 | 0.0177 |
| rs610516 | 82804963 | 0.0173 |
| rs668990 | 82807446 | 0.0185 |
| rs607544 | 82810884 | 0.0229 |
| rs662669 | 82818167 | 0.014 |
| rs603016 | 82820905 | 0.0127 |
| rs11658083 | 82821734 | 0.0194 |
| rs601997 | 82822508 | 0.014 |
| rs3744165 | 82832566 | 0.0243 |
| rs7225515 | 82837907 | 0.014 |
| rs4986113 | 82838360 | 0.0132 |
| rs11655723 | 82844028 | 0.0123 |
| rs7216324 | 82851589 | 0.0118 |
| rs11077948 | 82852057 | 0.0118 |
| rs9913679 | 82855022 | 0.0118 |
| rs2199111 | 82865327 | 0.0151 |
| rs9894705 | 82865882 | 0.0139 |
| rs7224427 | 82867331 | 0.0111 |
| rs4986122 | 82867624 | 0.0137 |
| rs12945629 | 82876571 | 0.05 |
| rs1317809 | 82888640 | 0.05 |
| rs4986131 | 82895920 | 0.0114 |
| rs2271918 | 82903330 | 0.0137 |
| rs2292971 | 82905981 | 0.0111 |
| rs4986136 | 82907243 | 0.0137 |
| rs8073910 | 82908551 | 0.0209 |
| rs3803768 | 82914152 | 0.0162 |
| rs8078148 | 82914909 | 0.0196 |
| rs8070801 | 82916226 | 0.0109 |
| rs8067859 | 82918990 | 0.0163 |
| rs1127986 | 82929368 | 0.0199 |
| rs3785514 | 82931029 | 0.0131 |
| rs2125479 | 82932386 | 0.0165 |
| rs7406464 | 82933584 | 0.017 |
| rs11869930 | 82935395 | 0.0167 |
| rs12940475 | 82944535 | 0.03 |
| rs9896263 | 82944865 | 0.0129 |
| rs1065144 | 82945581 | 0.0159 |
| rs3744157 | 82947423 | 0.0132 |
| rs7222745 | 82949377 | 0.0151 |
| rs8079082 | 82949683 | 0.0196 |
| rs12949939 | 82950625 | 0.0167 |
| rs2380173 | 82951281 | 0.0133 |
| rs6416855 | 82978629 | 0.0133 |
| rs9898649 | 82979429 | 0.0227 |
| rs9891371 | 82979952 | 0.0184 |
| rs7405583 | 82980689 | 0.0136 |
| rs9911294 | 82985540 | 0.0133 |
| rs9912207 | 82985610 | 0.0133 |
| rs9910113 | 82985734 | 0.0133 |
| rs9911830 | 82985809 | 0.0133 |
| rs7406848 | 82997333 | 0.0133 |
| rs7221423 | 83000756 | 0.0132 |
| rs9901757 | 83005455 | 0.0141 |
| rs751409 | 83007502 | 0.0143 |
| rs7405903 | 83011494 | 0.0143 |
| rs2168935 | 83011674 | 0.0147 |
| rs12603171 | 83014877 | 0.0164 |
| rs7213238 | 83019255 | 0.0159 |
| rs7208987 | 83027393 | 0.0159 |
| rs7222550 | 83027448 | 0.0158 |
| rs7406067 | 83029736 | 0.0141 |
| rs16940609 | 83035056 | 0.0153 |
| rs8065396 | 83037769 | 0.0153 |
| rs9908243 | 83046796 | 0.0164 |
| rs7224733 | 83046894 | 0.016 |
| rs10852789 | 83048410 | 0.0178 |
| rs1143006 | 83048511 | 0.0175 |
| rs7406119 | 83048753 | 0.016 |
| rs9911217 | 83064582 | 0.0167 |
| rs8077967 | 83087978 | 0.0117 |
| rs7502145 | 83090856 | 0.0221 |
| rs4986109 | 83091923 | 0.0158 |
